# Effective fabrication and characterization of eco-friendly nano particles composite for adsorption Cd (II) and Cu (II) ions from aqueous solutions using modelling studies

**DOI:** 10.1038/s41598-024-61050-1

**Published:** 2024-05-23

**Authors:** Mohammed Taha Moustafa Hussien Hamad, Sabah Ibrahim

**Affiliations:** Central Laboratory for Environmental Quality Monitoring, National Water Research Center, Shubra El Kheima, Egypt

**Keywords:** CS@Fe-PA, MgO@Pp, XPS study, Isotherm models, Biochemistry, Microbiology, Environmental sciences

## Abstract

The public health and environment are currently facing significant risks due to the discharge of industrial wastewater, which contains harmful heavy metals and other contaminants. Therefore, there is a pressing need for sustainable and innovative technologies to treat wastewater. The main objective of this research was to develop novel composites known as chitosan, *Padina pavonica,* Fe(III), and nano MgO incorporated onto pomegranate peel with the specific purpose of removing Cd (II) and Cu (II) ions from aqueous solutions. The characterization of these nanocomposites involved the utilization of several analytical methods, including Fourier-transform infrared spectroscopy, scanning electron microscopy, X-ray diffraction, thermal gravimetric analysis, and X-ray photoelectron spectroscopy. The efficiency of these nanocomposites was evaluated through batch mode experiments, investigating the impact of factors such as pH, initial concentration, contact time, and adsorbent dose on the adsorption of Cu(II) ions. The optimum conditions for the removal of ions were pH = 5 for Cu (II) and 6 for Cd (II), contact time: 120 min, adsorbent dosage: 0.2 g, initial metal ion concentration: 50 mg/L for each metal ion for the present study. The MgO@Pp demonstrated the highest removal efficiencies for Cu(II) and Cd(II) at 98.2% and 96.4%, respectively. In contrast, the CS@Fe-PA achieved removal efficiencies of 97.2% for Cu(II) and 89.2% for Cd(II). The modified MgO@Pp exhibited significantly higher total adsorption capacities for Cu(II) and Cd(II) at 333.3 and 200 mg/g, respectively, compared to CS@Fe-PA, which had capacities of 250 and 142 mg/g, respectively. The adsorption of Cd (II) and Cu (II) ions by MgO@Pp was found to be a spontaneous process. The R^2^ values obtained using the Freundlich and Redlich-Peterson models were the highest for the MgO@Pp composite, with values of 0.99, 0.988, 0.987, and 0.994, respectively, for Cu (II) and Cd (II). The pseudo-second-order equation was determined to be the best-fit kinetic model for this process. Reusability experiments confirmed that the adsorbents can be utilized for up to four regeneration cycles. Based on the findings of this study, MgO @ Pp is the most promising alternative and could be instrumental in developing strategies to address existing environmental pollution through adsorption.

## Introduction

The global water crisis is worsening, exacerbated by factors such as industrialization, climate change, and urbanization. These factors contribute to the production of hazardous materials and the increasing demand for water due to population growth and development^1^. Consequently, large amounts of wastewater are being released into the environment without proper reuse, leading to further contamination of our water resources^2^. One of the critical issues is the presence of heavy metal contaminants in wastewater, which poses a significant threat to human health. These pollutants can be found in industrial wastewater effluents and contaminated urban in the soil and can cause severe health issues when exposed to them^3^. In the long term, heavy metal toxicity can have a significant negative impact on both humans and the environment^4^.

To address this crisis, one promising strategy is the recycling of contaminated wastewater into clean water for agricultural and industrial purposes. Implementing this strategy can help reduce the misuse of natural water resources and prevent further environmental damage^5^. According to the World Health Organization (WHO), water scarcity is a looming global issue that could potentially affect up to 4 billion people by 2050^6^. While heavy metals like cadmium are essential for life in small amounts, they can be poisonous if present in larger amounts in the environment. Humans are particularly susceptible to cadmium poisoning, and even small amounts of it can cause long-term health complications such as liver and lung damage, increased blood pressure, impaired renal function, birth defects, and cancer^7^. Cadmium is an especially hazardous heavy metal, and its essential limit for plants is between 5 and 30 µg per gram, while the usual content of cadmium in the soil is around one microgram per gram^8^. The human body can typically absorb only about 0.001 mg of cadmium per kilogram of body weight per day^9^. Furthermore, the accumulation of heavy metals in land-dwelling animals can diminish their competitiveness, viability, and population size, ultimately leading to ecological disturbances^10^. Copper is a heavy metal that is both essential and highly toxic in high doses. As a result, it is crucial to remove such metal ions from aquatic environments to protect biodiversity, ecosystems, and people^11^. A comprehensive strategy should be developed to reduce the risks of heavy metal contamination and ensure the safety and sustainability of our environment^13^. To achieve this, a variety of remediation methods have been developed. These include physical, chemical, and biological approaches. Physical remediation methods involve the use of granular activated carbon, membrane separation, and supercritical fluid extraction, while chemical remediation encompasses chemical precipitation^13^, Fenton oxidation^14^, electrocoagulation, and electrolysis^15^. These methods are effective in removing a broad spectrum of contaminants, but they are often costly, require a lot of energy and additional chemicals, and can even cause secondary pollution. Hence, it is imperative to devise effective and innovative technologies that can be combined with existing ones to efficiently and cost-effectively remove heavy metals from wastewater effluents^16^. In recent years, new nano adsorbents utilizing nanomaterials are being developed. Nanomaterials offer exceptional properties such as high specific surface area, minimal generation of flocculants, high adsorption capacity, rapid kinetics, and the ability to be regenerated and reused for multiple cycles^17^. Numerous studies have presented evidence supporting the practical application of nanotechnology in remediating metal pollution. This includes the use of molecularly imprinted polymers (MIP), nanocatalysts, bioactive nanoparticles, nanostructured catalytic membranes, nanosorbents, and carbon nanotubes for removing pollutants^18^. Chitosan is a natural biopolymer that is biocompatible, chemically stable, and biodegradable with nontoxic degradation products. It has shown great potential as an adsorbent for heavy metal ions and dyes in decontamination applications. The abundance of amino and hydroxyl groups in chitosan plays a crucial role in facilitating effective sorption processes^19^. Metal oxide nanoparticles, including magnesium oxide (MgO), have emerged as up-and-coming candidates for large-scale wastewater treatment in industrial settings. These nanoparticles possess advantageous characteristics such as cost-effectiveness, exceptional adsorption capacity, unique chemical, functional, and physical properties, small bandgap energy (2.4–2.8 eV), stable physicochemical properties, and strong photo corrosion stability in aqueous solution as well as stable recyclability potentials ease of separation, and improved stability. These properties enable them to remove heavy metals from wastewater efficiently^20^. The research on marine macro-algae exhibited the highest efficiency in removing heavy metal ions in comparison to other adsorbents studied^21^. The potential of pomegranate peel as an adsorbent for eliminating diverse pollutants from aqueous solutions has been extensively researched. Several studies have demonstrated the high adsorption capacity of pomegranate peel, which is primarily attributed to the presence of functional groups such as carboxyl, hydroxyl, and phenolic groups. These groups are capable of interacting with the adsorbate via electrostatic attraction, hydrogen bonding, and other mechanisms^22,23^.investigated the use of pomegranate peel as an adsorbent for the removal of lead and Cu^2+^ ions from aqueous solutions. The study found that pomegranate peel exhibited a high adsorption capacity for lead and copper ions, with a maximum removal efficiency of 90.2% and 80%, respectively. For example, *Padina pavonica* has shown a high uptake capacity of 2.74 mg/g for Cu^2+^ and 24.93 mg/g Pb(II) ions at pH 6.30, respectively^24^. The combination of chitosan and *Padina pavonica* as an adsorbent material has many advantages due to their low cost and eco-friendly nature, as well as their wide availability. Another research group^17^, found that the coating of chitosan on algae (BBC) enhanced the removal of Cr(VI) and Pb (II) from10 to 25.8% and 22.9 to 57.6%, respectively, compared to the unmodified algae. Innovatively combining synthetic nanoparticles, consisting of MgO with pomegranate peels and chitosan-coated Fe(III) *Padina pavonica*, holds great promise as an affordable and effective approach for eliminating heavy metals from wastewater. This could have a positive impact on safeguarding the environment and promoting human health. Prior research has established that effective loading of magnesium oxide onto pomegranate peels can increase the number of sorption sites and enhance the nanocomposite electrostatic attraction ability, anion exchange capacity, and precipitation. As a result, MgO- pomegranate peel composites have shown a high affinity for anionic contaminants in aqueous solutions, as confirmed by recent studies^25^. In this study, our objective was to create a multifunctional material by incorporating nanosized MgO into impregnated pomegranate peels and chitosan combined with Fe(III) *Padina pavonica*. This composite is aimed at serving as an effective adsorbent with high adsorption capacity and rapid metal ion removal in aqueous media. Additionally, the fabrication method we proposed is environmentally friendly, cost-effective, time-saving, and free from harmful chemicals. The nanosized MgO-impregnated pomegranate peels, chitosan, and Fe(III) *Padina pavonica* used in the composite are derived from natural sources, ensuring their safety for both humans and the environment. The synthesized adsorbent composites were thoroughly characterized using various techniques, including XRD, XPS, TEM, SEM/EDX, and FTIR. Suitable isotherm and kinetic models were employed to predict the adsorption process. The reusability and regenerative properties of the developed adsorbents were extensively investigated over multiple cycles.

## Material and methods

### Chemicals

Analytical grades of CuSO_4_.5H_2_O, CdSO_4_.H_2_O and MgCl_2_·6H_2_O were obtained from Merck, Egypt. Iron (III) chloride hexahydrate was purchased from Sigma-Aldrich Co., Ltd. Chitosan (80–100 mesh) 90% acetylation degree, glutaraldehyde solution and sodium hydroxide (NaOH) were ordered from Pioneers for Chemical Co., Ltd., Egypt.

### Synthesis

#### Synthesis of MgO nanoparticles by plant extract

The synthesis of MgO nanoparticles involves a few steps that must be followed in order to produce the nanoparticles. The first step is to prepare a feedstock consisting of dried pomegranate peels at 100 °C, which should be ground in a ball mill and followed by sieving to achieve particle sizes of 100 mm. The second step involves the preparation of Mulberry leaf extract. The Mulberry leaves were washed thoroughly using distilled water to eliminate dust and dirt. The plant collection and use were in accordance with all the relevant guidelines. The mulberry leaves and pomegranate peel used in this study were purchased from the supermarket. Eleven grams of leaves were boiled in 120 mL of deionized water at 100 °C for 5 min. After that, the solution was allowed to cool, filtered, and stored at a temperature of 4 °C. In the third step, the *Padina pavonica* underwent activation by being treated with a solution of pure CaCl2 and H2O in a ratio of 2:4 (v/v) for 24 h. Subsequently, the *Padina pavonica* pulp was once again crushed and then subjected to lyophilization for 24 h to ensure complete drying of the *Padina pavonica*^26^. In the fourth step, mix 50 L of a 1 M solution of MgCl_2_·6H_2_O with 20 mL of Mulberry leaf extract. Slowly add 1 M NaOH drop by drop while continuously stirring the mixture using a magnetic stirrer for 2 h. Additionally, add 4 g of pomegranate peels to the flask. The mixture must then be stirred for 6 h at a temperature of 80 °C. After this, the solid–liquid dispersion must be centrifuged at 4000 rpm for 14 min in order to remove any excess MgCl_2_ 6H_2_O and residual organic molecules. It is necessary to rinse the residue with deionized water and subsequently dry it in an oven at a temperature of 70 °C for 2 h. Ultimately, to acquire the magnesium oxide nanoparticles (MgONPs), it is essential to subject them to a calcination process in a furnace, which involves heating at 500 °C for 3 h. This process results in the formation of the desired MgONPs. The MgONPs should then be sonicated at 40 °C for 1 h in an ultrasonic cleaner (Daihan-Scientific) in order to disperse the particles^27^.

#### Manufacture of CS@Fe-PA sample collection and bleaching treatment

*Padina pavonica* (L.) is an essential type of marine macroalgae found in the coastal zone of the Red Sea in Egypt. During the spring season, samples of this alga were collected from the coastal zone of Mousa coast, Ras Sedr government, Egypt. These samples had flattened fan-shaped thalli up to 15 cm in size and were typically found growing on submerged rocks up to 50 cm in depth. The first step in the preparation of *Padina pavonica* involved washing the samples under running tap water to remove any surface debris. The marine macro-algae were then air-dried for about 30 min and oven-dried at 50 °C. The *Padina pavonica* was activated through treatment with a solution comprising pure CaCl_2_ and H_2_O at a ratio of 2:4 (v/v) for 24 h. The dried biomass was then crushed with a blender and sieved with a 100 μm sieve before being stored in an airtight container at room temperature. The second step involved the deposition of Fe(III) particles onto the *Padina pavonica* (L.) surface using a modified method^21^. Eight grams of the prepared alga were submerged in a 250 mL solution of FeCl_3_·6H_2_O with a 0.1 mol/L concentration for 120 min, stirring continuously.

After this, the *Padina pavonica* underwent a drying process in an oven set at 70 °C for 24 h. Subsequently, it was thoroughly rinsed multiple times with deionized water to eliminate any impurities before undergoing another round of oven-drying, rendering it ready for experimentation. Finally, a coating process was employed to synthesize a thin layer of chitosan on the surface of *Padina pavonica*. The process began by dissolving 3 grams of chitosan in 250 mL of 2.5% (v/v) acetic acid. Subsequently, 5 grams of the prepared *Padina pavonica* were introduced into the solution. The mixture was stirred continuously for 120 min. Next, 1.3% NaOH was added drop by drop to the homogeneous mixture until the pH level escalated to 9.5, which is the optimal condition for chitosan coating. The mixture was then allowed to sit at room temperature for 24 h, after which it was washed with DI water to eliminate any excess NaOH. The fourth step involved incorporating 200 mL of a 5% OHC(CH2)3CHO-solution and allowing the mixture to cure through cross-linking for 2.5 h. The C_2_H_6_O solution underwent a washing process, followed by filtration and subsequent drying in a desiccator to yield CS@Fe-PA beads^28^.

### Preparation of Cu^2+^ and Cd^2+^ ions solutions

Initially, the appropriate quantities of CuSO_4_ 5H_2_O and CdSO_4_ 4H_2_O were dissolved in 1-liter volumetric flasks to prepare stock solutions with a concentration of 1000 mg/L for Cu^2+^ and Cd^2+^ ions.

### Zero point of charge of nano composites

To ascertain the pH_ZPC_ values of MgO@Pp and CS@Fe-PA composites, a quantity of 0.2 grams of each sample was added to a 50 mL solution of 0.1 M NaCl, with an initial pH range spanning from 2 to 11. The mixture was vigorously shaken for 48 hours. Subsequently, the sample was separated from the solution, and the equilibrium pH values of the solution were measured. The discrepancy between the initial and equilibrium pH values was graphed against the initial pH of the solution. The point at which the graph intersected the x-axis was recognized as the pH_ZPC_ value, as described in the study by^29^.

## Instrumentation

FTIR Spectrum (Model: Bruker-VERTEX 80 V) (400–4000 cm^−1^) was utilized to get FTIR spectra of adsorbents. The particle size of the nanoparticles was determined using a transmission electron microscope (model JEOL-JEM-2100 FS, Japan) operated at 200 kV. These adsorbents were imaged by a scanning electron microscope (SEM) (Model: Quanta 250) attached to an EDX unit (Energy Dispersive X-ray Analyses) with an accelerating voltage of 30K. XRD analysis was carried out using Bruker D8 with radiation Cu Kα (λ = 1.54 Å). The pH measurements were performed using a digital pH meter (Multi-9620IDS-pH-meter, WTW, Germany). Metal concentrations were determined using Inductively Coupled Plasma Mass Spectrometry (ICP-MS) on the Perkin Elmer Sciex ELAN 9000. Thermo gravimetric and differential thermal analyses (TGA) were carried out using LABSYS evo TGA–DSC-analyzer units (Setaram- French). X-ray photoelectron spectroscopy (XPS) was performed on a K-ALPHA + XPS System (Thermo Fisher Scientific, U.SA.) with an X-ray Al- Kα 10 to 13,506 eV).

### Adsorption studies

Several rounds of batch tests were carried out to evaluate the adsorption capacities of CS@Fe-PA and MgO-impregnated pomegranate peel composites for Cu^2+^ and Cd^2+^ ions. The experiments were carried out in 250 mL flasks, with each flask containing 100 mL of metal solution at varying concentrations (30–100 ppm) and adsorbent dosages (0.2–1.2 g). The mixtures were placed on a thermal shaker and agitated for 120 min at a speed of 210 rpm and a temperature of 25 °C. The initial concentration of Cu^2+^ and Cd^2+^ metals was optimized within the range of 30–100 ppm at a pH of 5, a temperature of 25 °C, and an adsorbent dosage of 0.2 g. Likewise, the impact of pH on the activity of the adsorbent was investigated by examining various pH levels (3.0, 5.0, and 6.0) while keeping the adsorptive concentration (50 ppm), contact time (120 min) and adsorbent dosage (0.2 g) constant. Additionally, the adsorbent dosage was investigated by conducting experiments at different dosage levels ranging from 0.2 to 1.2 g/L while maintaining a constant pH of 5 and adsorptive concentration of 50 ppm. Lastly, contact time optimization was explored by varying the contact time from 15 to 120 min while keeping the pH, adsorptive concentration, and adsorbent dosage constant at pH 5, 50 ppm, and 0.  g, respectively. The removal efficiencies (RE%) of the adsorbents were calculated using the following equation:

Where A_0_ initial represents the initial concentration of Cu^2+^ and Cd^2+^ ions in the solution (mg/L) and A_f_ final represents the residual concentration of Cu^2+^ and Cd^2+^ metal ions in the solution (mg/L).

1 $$\mathrm{\%RE}=\frac{{{\text{A}}}_{{\text{o}}}-{{\text{A}}}_{{\text{f}}}}{{{\text{A}}}_{0}}\times 100$$

The adsorption capacity (qe, mg/g) at equilibrium was determined using Eq. (2):

2 $${\text{qe}}=\frac{({{\text{C}}}_{{\text{i}}}-{{\text{C}}}_{{\text{e}}})}{{\text{M}}}{\text{V}}$$

The concentrations of metal ions in the initial solution (Ci) and at equilibrium (Ce) are expressed in mg/L. The volume of the solution (V) is measured in liters, and the mass of the adsorbent (M) is measured in grams.

### Adsorption kinetics

To investigate the kinetic adsorption process, experiments were performed on 50 ml flasks. A total of 0.2 g of CS@Fe-PA and MgO@Pp composites were added to a solution containing 50 ppm of Cu^2+^ and Cd^2+^. The flasks were placed in a shaker and agitated for various time intervals ranging from 15 min to 2 h. Following the agitation, the mixtures of Cu^2+^ and Cd^2+^with the sorbents were subjected to centrifugation at 4000 rpm for 10 min and subsequently filtered through a 0.45 μm filter. The concentration of total Cu^2+^ and Cd^2+^in the filtered solutions was then measured. The kinetics of Cu^2+^ and Cd^2+^ sorption onto the two sorbents were modeled using different approaches, including first-order^30^, pseudo- second order^31^, Intra-particle diffusion and Elovich^16^. In the equation, K_1_ represents the rate constant for pseudo-1st-order kinetics (min^-1^), while qe and qt denote the equilibrium and time-dependent amounts of adsorbed metal ions (mg/g), respectively. When the process deviates from linearity, it is referred to as the (PSO) model, which is characterized by the rate constant k^2^ (mg/g min)^17^.

3 $${\text{log}}\left({{\text{q}}}_{{\text{e}}}- {{\text{q}}}_{{\text{t}}}\right)={{\text{logq}}}_{{\text{e}}}-\left(\frac{{{\text{k}}}_{1}}{2.303}\right){\text{t}}$$

4 $$\frac{{\text{t}}}{{{\text{q}}}_{{\text{t}}}}= \frac{1}{{{\text{k}}}_{2 }{{\text{q}}}_{{\text{e}}}^{2}}+ \frac{1}{{{\text{q}}}_{{\text{e}}}}\mathrm{ t}$$

5 $${{\text{q}}}_{{\text{e}}}={{\text{K}}}_{{\text{P}}}{{\text{t}}}^\frac{1}{2}+{\text{Ci}}\left(\upbeta \right)$$

6 $${{\text{q}}}_{{\text{t}}=\frac{1}{\mathrm{\alpha }}{\text{ln}}\left(1+\mathrm{\alpha },\mathrm{ \beta t}\right) }$$

### Adsorption isotherms

The adsorption experiments were performed employing a batch system. In each experiment, 0.2 g of the respective adsorbents was mixed with Cu^2+^ and Cd^2+^solutions ranging from 30 to 100 mgL^−1^ in 200 mL flasks. The mixtures were agitated using a shaker for a predetermined equilibrium adsorption time of 2 h. After the agitation, the samples were subjected to centrifugation, filtration, and measurement of Cu^2+^ and Cd^2+^concentrations, following the same procedure described in the adsorption kinetics section. The obtained sorption data were analyzed using Langmuir, Freundlich^32^ and Dubinin-Radushkevich, Redlich–Peterson, and Temkin isotherm models and thermodynamic dynamics^13^.

7 $$\frac{{{\text{C}}}_{{\text{e}}}}{{{\text{q}}}_{{\text{e}}}}= \frac{{{\text{C}}}_{{\text{e}}}}{{{\text{Q}}}_{{\text{max}}}} + \frac{1}{{{\text{Q}}}_{{\text{max}}}{{\text{K}}}_{{\text{L}}}}$$

8 $${{\text{R}}}_{{\text{L}}}=\frac{1}{1+{{\text{K}}}_{{\text{L}}}{{\text{C}}}_{\circ }}$$

9 $${{\text{lnq}}}_{{\text{e}}}={{\text{lnK}}}_{{\text{F}}}+\frac{1}{{\text{n}}}{{\text{lnC}}}_{{\text{e}}}$$

10 $$ q_{e} = q_{m} \exp ( - K_{ad } \varepsilon^{2} ) $$

11 $$ \varepsilon = {\text{RTln}}\left\lfloor {1 + \frac{1}{{{\text{C}}_{{\text{e}}} }}} \right\rfloor $$

12 $$ E = \left\lfloor {\frac{1}{{\sqrt 2 K_{ad} }}} \right\rfloor $$

13 $$ {\text{Qe}} = {\text{ B ln AT }} + ({\text{RT}}/{\text{b}}){\text{ln Ce}} $$

14 $$ \ln \left\lfloor {KR\frac{{C_{e} }}{{q_{e} }} - 1} \right\rfloor = \ln \alpha R + \beta \ln C_{e} $$

15 $${{\text{lnK}}}_{{\text{L}}}= \frac{{-\Delta {\text{H}}}^{\circ }}{{\text{RT}}}+ \frac{{\Delta {\text{S}}}^{\circ }}{{\text{R}}}$$

16 $$\Delta {\text{G}}^\circ = \Delta {\text{H}}^\circ -{\text{T}}\Delta {\text{S}}^\circ $$

### Adsorbent reusability

The economic viability and reusability of CS@Fe-PA and MgO@Pp composites were assessed by regenerating the spent nanocomposite adsorbents and testing their effectiveness in practical applications. Typically, 1 g of CS@Fe-PA and MgO@Pp composites were subjected to shaking with 50 mg/L Cu^2+^ and Cd^2+^ solutions for 2 h using an orbital shaker. Once equilibrium was reached, the Cu^2+^ and Cd^2+^loaded CS@Fe-PA and MgO@Pp composites were dried and immersed in 50 mL of 0.1 M HCl, followed by shaking for 2 h at room temperature and drying. Following each treatment, the adsorbent material was separated from the Cu^2+^ and Cd^2+^solutions, rinsed with distilled water, and subsequently soaked in distilled water overnight. Following filtration, the solid residue underwent another cycle of agitation with a new 200 mL solution of Cu^2+^ and Cd^2+^ (50 mg/L). This procedure was repeated for up to 4 consecutive cycles, and the removal efficiency of Cu^2+^ and Cd^2+^was calculated after each cycle.

## Results and discussion

### Characterization analysis of nanocomposites

#### TEM and SEM analyses

**Figure 1:**
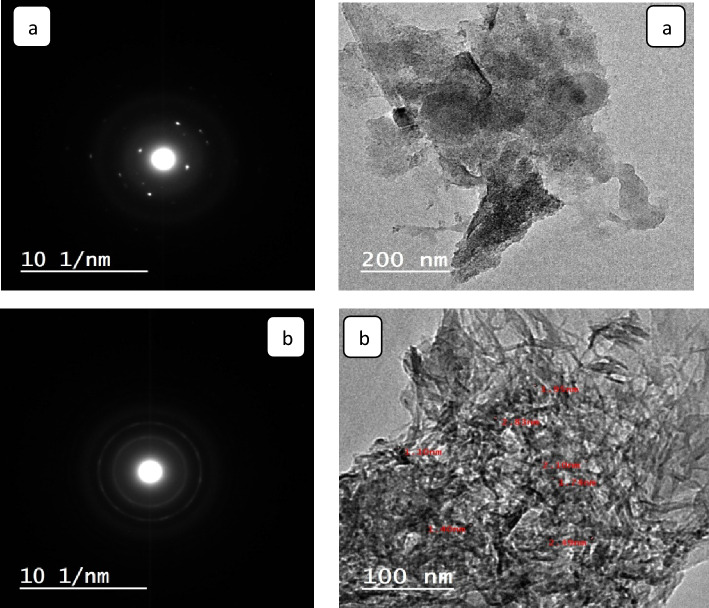
TEM images of (**a**) CS@Fe-PA and (**b**)MgO@Pp composites.

**Figure 2:**
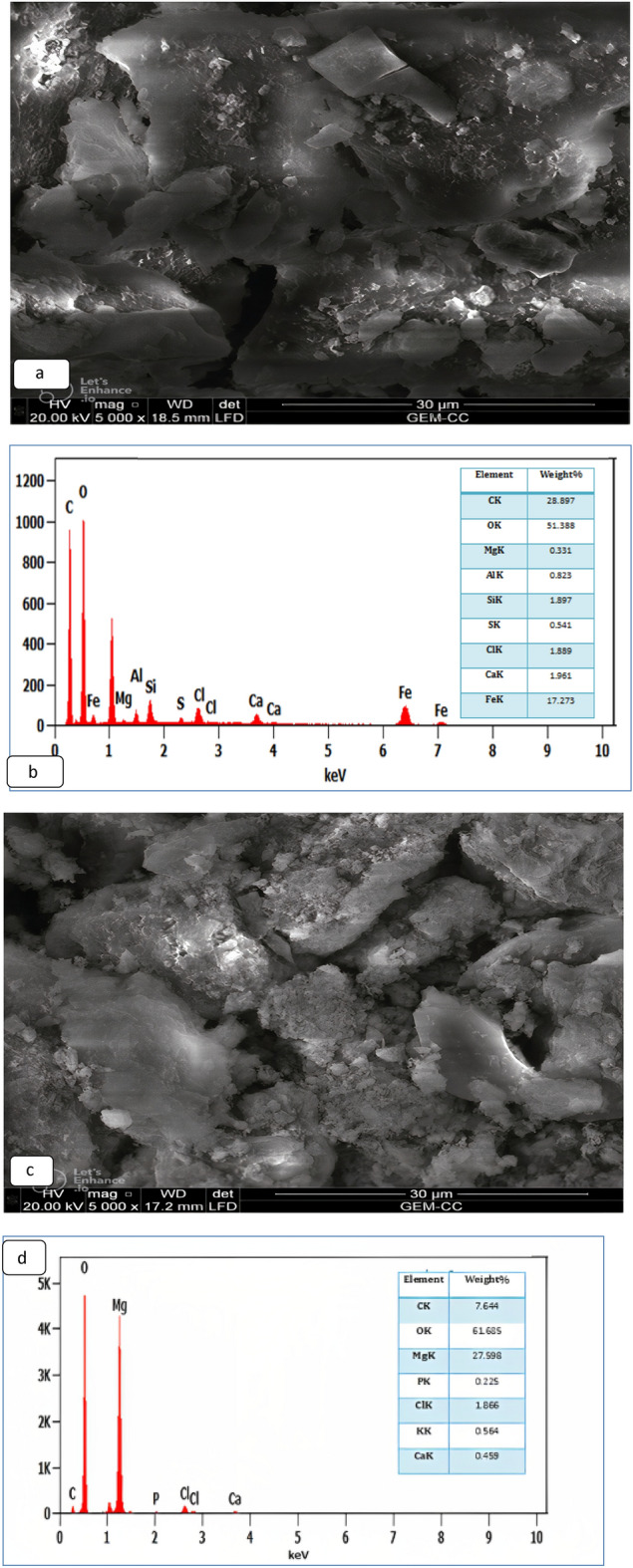
SEM images of CS@Fe-PA (**a**, **b**) and (**c**, **d**) MgO@Pp composites.

A transmission electron microscope (TEM) was utilized to assess the size of the CS@Fe-PA and MgO@Pp composites. The synthesized CS@Fe-PA and MgO@Pp composites exhibited a maximum size of approximately 0.58  and 1.59 nm, respectively. TEM images clearly displayed the uniform distribution of MgO nanoparticles on the surface of the pomegranate peels. The graph from the TEM analysis indicated that the MgO nanoparticles were enveloped by a significant amount of pomegranate peels, resulting in a low percentage of Mg by weight in the MgO@Pp composite. Conversely, the TEM photograph revealed that the CS@Fe-PA particles predominantly consisted of aggregates of α-FeOOH (depicted as black areas) dispersed throughout the chitosan matrix, Fig. 1a,b. SEM and EDS analyses were conducted to compare the microstructure and surface morphology disparities between the CS@Fe-PA and MgO@Pp composites. The SEM images presented in Fig. 2a,c exhibited distinct morphological structures and varying particle sizes, confirming the successful cross-linking of chitosan with Fe(III) and the surface modification of algae with glutaraldehyde. The divergence in particle size and morphology resulted in variations in the physical and chemical properties of the nano-sorbents, which influenced. The physical and chemical characteristics of the nano-sorbents fluctuate due to variances in particle size and morphology, which have an impact on the sorption process and the availability of active sites for the adsorption of heavy metals. The SEM image shows a rough and porous surface with irregular shapes and particles of different sizes. The particles are densely packed, and there is no visible agglomeration, indicating a uniform distribution of MgO and Fe(III) in the composite. The surface characteristics and structure of the composite suggest that MgO has been well impregnated into the pomegranate peel, chitosan, and *Padina pavonica* have been well integrated into the Fe(III) particle matrix resulting in a highly porous and rough surface that can enhance the composite's adsorption capacity. The EDX spectra of CS@Fe-PA presented in Fig. 2b,c showed peaks corresponding to C, O, Cl, Mg, Al, Si, Ca, Fe, and S. These caused the loading of Fe (III) onto the surface of CS. C and O elements contribute 28.3% and 51.3%, respectively. This indicates a higher active carbon content in CS@Fe-PA, which can be attributed to the macro-algae cell wall, which possesses diverse functional groups such as phosphoryl, carboxyl, amino, hydroxyl, and sulfhydryl. This is further supported by the use of glutaraldehyde for cross-linking purposes. Meanwhile, the EDX analysis Fig. 2d revealed that the significant elements of the MgO@Pp composite were O, Mg, C, and Cl elements, contributing 59.6, 22.8, 6.9, and 9.12%, respectively. The more pronounced peaks for oxygen and carbon elements were attributed to the polyphenols, cellulose, and hemicellulose present in pomegranate peels. Additionally, the presence of Ca and Al elements originated from the pomegranate peel feedstock, as well as mulberry leaf extract. The presence of Mg confirmed the existence of MgO and indicated the successful incorporation of MgO into the pomegranate peel matrix. Moreover, the inclusion of base cations such as Ca and Mg could contribute to the adsorption of metal ions by the MgO@Pp composite, as suggested by previous studies^32^.

#### Fourier transforms infrared (FTIR) analysis

**Figure 3:**
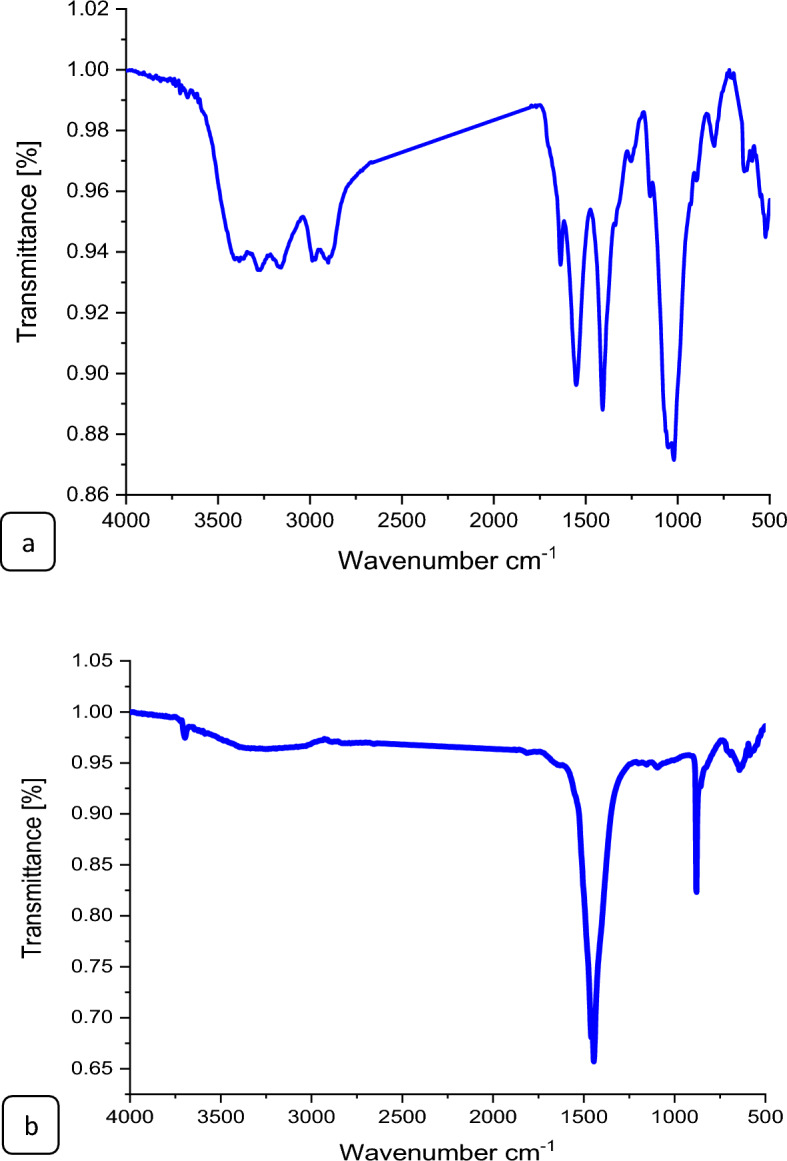
FTIR spectra of the CS@Fe-PA (**a**) and (**b**)MgO@Pp composites.

The FTIR spectrum of MgO@Pp composite exhibits several characteristic bands. Here's an interpretation of the prominent bands in the spectrum, 3697.3 cm^−1^, as shown in Fig. 3a: This broad and intense peak is attributed to the stretching vibration of O–H groups in the composite material, indicating the presence of hydroxyl groups. 3240.1 cm^−1^: This peak is also due to O–H stretching vibration but appears at a lower wavenumber than the previous peak^33^. It may arise from the presence of water molecules bound to the MgO surface or from aliphatic and phenolic hydroxyl groups in the pomegranate peels and extract plant 1445.1 cm^−1^: These peaks correspond to the bending vibrations of C–H bonds in the composite. They may arise from the methylene (–CH2–) groups in the pomegranate peels^34^.The deformation vibration of Mg–O–Mg, Si–O–Mg of Mg–O appeared at 1459 cm^−1^ and 410.1 cm^−1^^35^, indicating that MgO was loaded on pomegranate peels successfully.1094.2 cm^−1^: This peak corresponds to the stretching vibration of Si–O bonds in the MgO particles, indicating the presence of the MgO component. 880.2 cm^−1^: This peak is attributed to the stretching vibration of C–O bonds in the composite, indicating the presence of carboxyl and ester groups in the pomegranate peels^36^. 690.6 cm^−1^, 642.9 cm^−1^, and 599.1 cm^−1^: These peaks correspond to the bending vibrations of C–H bonds in the aromatic ring of lignin or other aromatic compounds in the pomegranate peels. 444.1 cm^−1^ and 419.2 cm^−1^: These peaks correspond to the stretching vibration of Mg–O bonds in the MgO@Pp composite^37^. In contrast, the FTIR spectrum of CS@Fe-PA exhibits characteristic bands of the CS@Fe-PA shows distinctive bands that provide valuable information about its chemical composition and structure Fig. 3b. The bands observed at 3852.92, 3743.96, 3730.28, 3706.32, 3665.49, 3625.69, 3607.11, and 3384.11 cm^−1^ correspond to the stretching vibration of O–H and N–H bonds, indicating the presence of chitosan and water molecules in the composite structure. 2987.62 cm^−1^: This peak corresponds to the stretching vibration of C–H groups in the *Padina pavonica* component of the composite. 2901.28 cm^−1^: This peak corresponds to the stretching vibration of C–H groups in the chitosan component of the composite. 1637.02 cm^−1^: This peak corresponds to the amide I band in the chitosan component of the composite, indicating the presence of the C=O stretching vibration and N–H bending vibration^38^. 1551.95 cm^-1^: The observed peak corresponds to the amide II band in the chitosan component of the composite, suggesting the presence of N–H bending vibration and C–N stretching vibration^39^. 1408.31 cm^−1^: This peak corresponds to the stretching vibration of COO– groups in the chitosan component of the composite^40^. 1253.37 cm^−1^: This peak corresponds to the stretching vibration of C–O groups in the *Padina pavonica* component of the composite. 1150.04 cm^−1^, 1050.95 cm^−1^: These peaks correspond to the stretching vibration of Fe–OH bonds in the Fe(III) component of the composite. 1020.84 cm^−1^: This peak corresponds to the stretching vibration of C–O–C groups in the chitosan component of the composite.897.84 cm^−1^: This peak corresponds to the bending vibration of C–H groups in the *Padina pavonica* component of the composite. 801.48 cm^−1^, 701.48 cm^−1^: The observed peaks at 637.57 cm^−1^ in the FTIR spectrum correspond to the bending vibration of Fe–OH bonds present in the composite's Fe(III)-chitosan structure. This peak corresponds to the bending vibration of N–H groups in the chitosan component of the composite. 594.69 cm^−1^: This peak corresponds to the bending vibration of C–H groups in the biocarb component of the composite. 522.66–469.93 cm^−1^: The presence of the Fe–O–CHT group was validated by the characteristic peaks observed in the spectrum, specifically in the range of 522.66–469.93 cm^−1^. This provides evidence that the hydroxyl (OH) groups of chitosan actively participate in the complexation process with ferric ions, as indicated by^18^. This finding further supports the notion of a robust interaction between the loaded metal oxides and the amide groups within the chitosan matrix^41^.

#### Crystallographic characteristics

**Figure 4:**
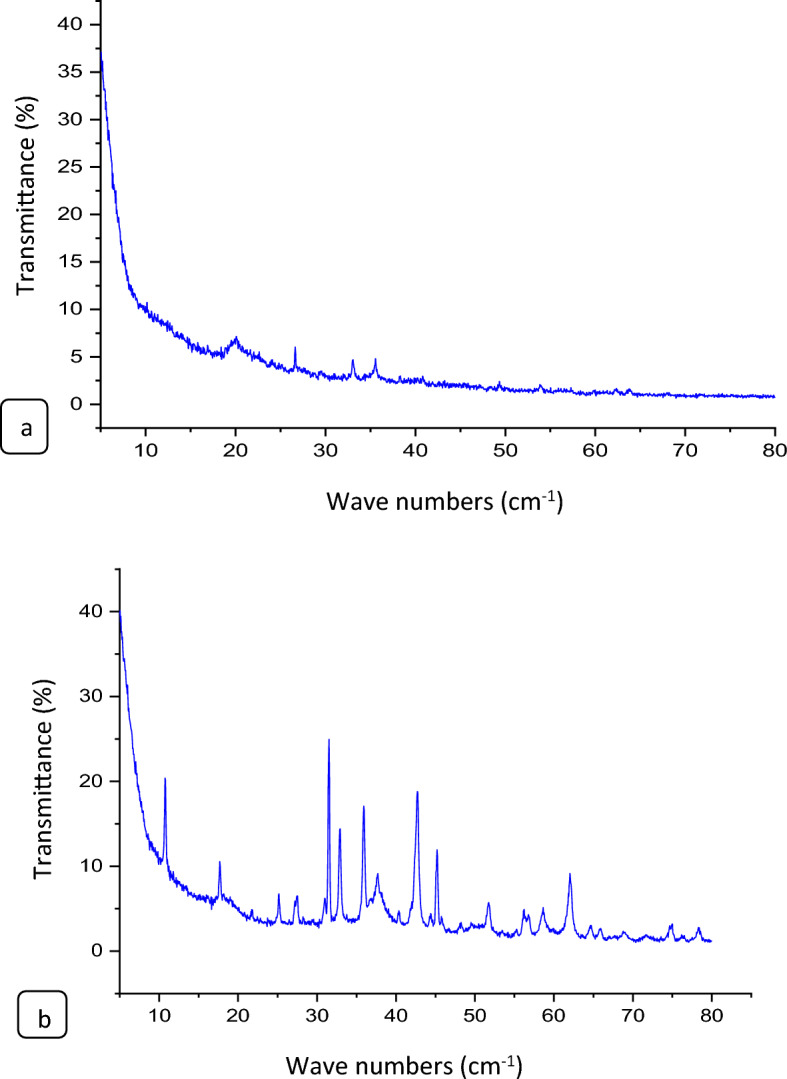
XRD results of the CS@Fe-PA (**a**) and (**b**)MgO@Pp composites.

The X-ray diffraction (XRD) result obtained from CS@Fe-PA (shown in Fig. 4a) displays multiple diffraction peaks, which are located at 2θ values of 20.101° 24.068° 26.629° 29.508 (belong to chitosan),° 33.037° 35.550° 40.823° 49.343° 53.930° 62.293° 63.79 (belong to Fe(III) on chitosan). These peaks are associated with the crystal planes (110), (120), (130), (101), (200), (230), (240), and (640), respectively. These crystal planes are similar to those found in goethite minerals (ICDD PDF (No.69–221–1653). Based on this outcome, we can deduce that the primary form of Fe(III) found in the prepared beads is α-FeOOH^42^. The findings suggest that the NH2 and OH groups of CHT interact with Fe ions, supporting the favorable compatibility between the polymer matrix of CS@Fe-PA. Figure 4b shows the XRD pattern and the crystalline properties of the synthesized MgO@Pp composite. As the Fig 4b. demonstrates the emergence of five new diffraction peaks at 2θ approximately 37.677° 42.709° 62.042° 74.940° 76.289° 78.317 corresponds to the (111), (202),(222), (311), and (222) planes (JCPDS No. 96-101-1174), which reveals the successful decomposition of MgO crystallite onto pomegranate peels^43,44^. Diffraction peaks appeared at 2θ around 10.77° (1 1 0), 17.6° (0 0 2), 25.1° (1 2 0), 27.4° (1 0 3), 30.99° (200), and 35.9° (123) for pure pomegranate peels can be attributed to the reflection of carbon based on JCPDS file No. 96-101-1022, indicating the presence of amorphous carbon in pomegranate peels, as confirmed by^18^. However, in the MgO impregnated pomegranate peels composite, the intensity of diffraction peaks associated with amorphous carbon noticeably decreases due to the high loading of MgO onto the pomegranate peels. The broad peak observed in the spectrum can be ascribed to the existence of organic compounds derived from Pomegranate peels feedstock and Mulberries leaves extract, which play a role in coating and stabilizing the MgO within the Pomegranate peels matrix, as suggested by^27^. The peaks shown at angles 10.77 and 25.1 are related to carbon^45^.

#### Thermal gravimetric (TGA) analysis for characterization of nano composites

**Figure 5:**
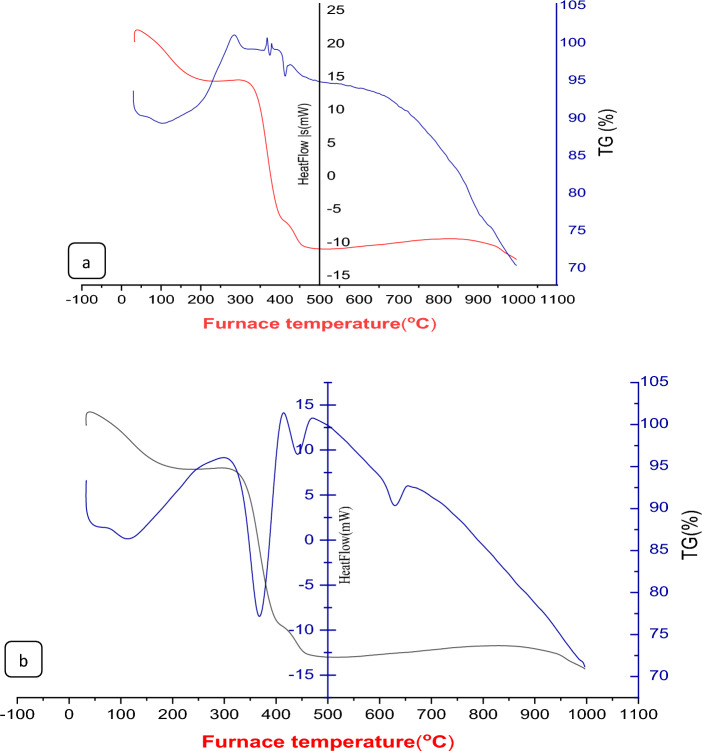
TGA spectra of (**a**) CS@Fe-PA and (**b**) MgO@Pp composites.

The thermograms of CS@Fe-PA and MgO@Pp composite adsorbents are represented in Fig. 5a,b. The thermal analysis of the CS@Fe-PA composite reveals three distinct stages of decomposition. The initial stage, occurring in the temperature range of 52-173 °C, exhibits a mass loss of 3.79%, corresponding to the release of hydrated water molecules. The second stage, with a weight loss of 27.83% within the temperature range of 186-350 °C, is attributed to the decomposition of the -NH_2_ and -CH_2_OH groups of chitosan^41^, the decline in wt% loss of CS@Fe-PA composite at temperatures 356–537 and 608–993 °C suggests significant thermal stability introduced in the CS@Fe-PA composite structure by chitosan^46^. In contrast, the thermal analysis curve of the MgO@Pp composite shows 40–227, 240–410, and 411–495 °C, with the percentage loss values corresponding to 6.6, 18.6, 8.376 and 3.68%, respectively. The first stage of thermal degradation primarily involves the desorption of water molecules, while the subsequent stages are associated with the partial decomposition of the MgO@Pp composite. The total weight loss percentage of this sorbent is measured at 28.88%, which is lower than that of the CS@Fe-PA composite (50.2%).

#### XPS analysis of nano composites

**Figure 6:**
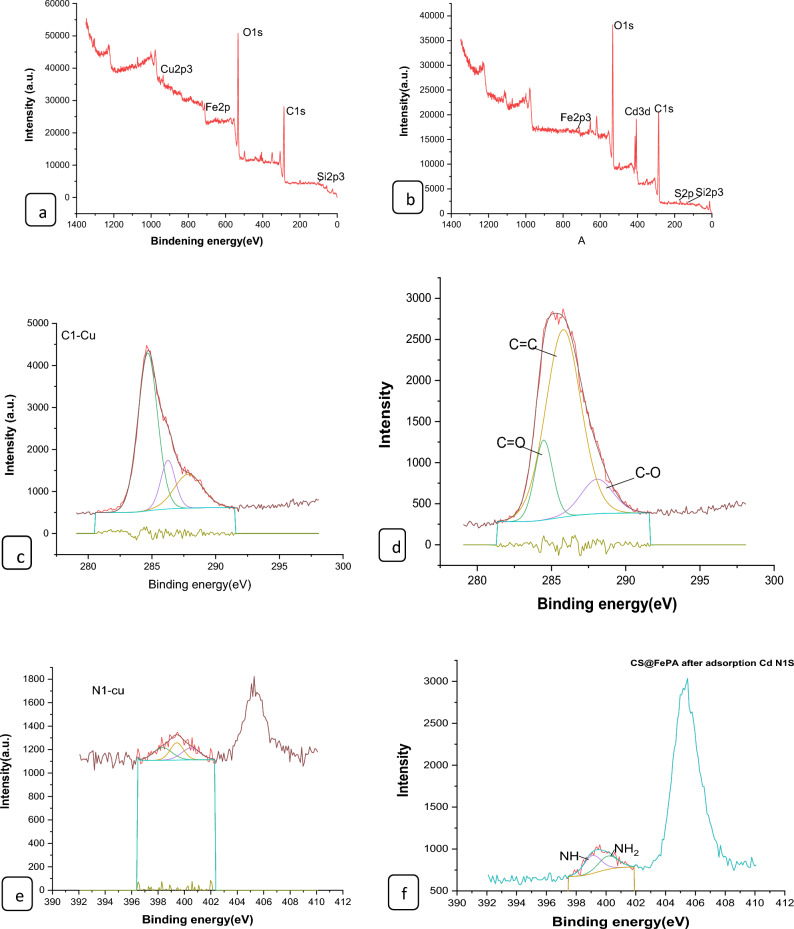

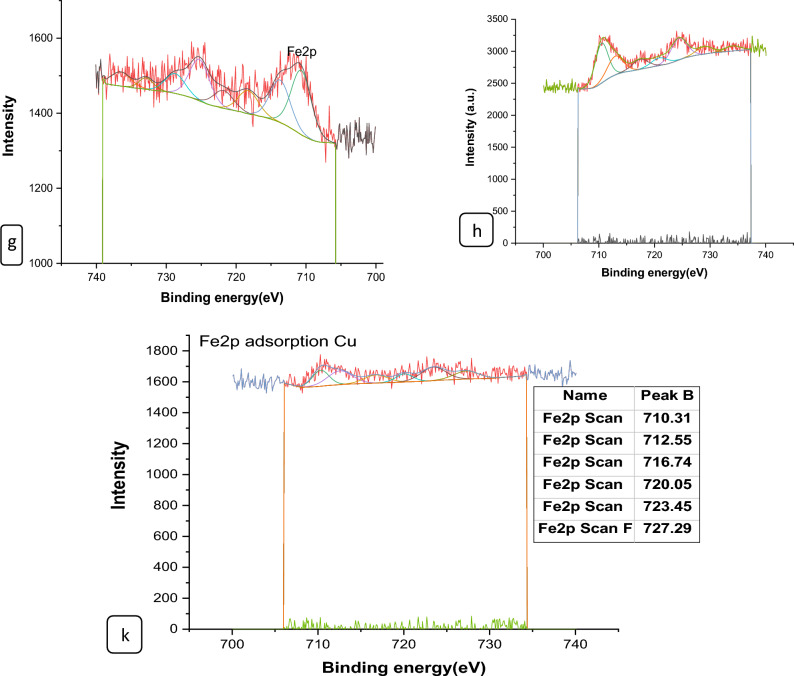
High‐resolution XPS spectra of pure CS@Fe composite: (**a**) C 1s(**a**–**c**),and N1(**d**–**g**, **k**) before and after Fe2p.

**Figure 7:**
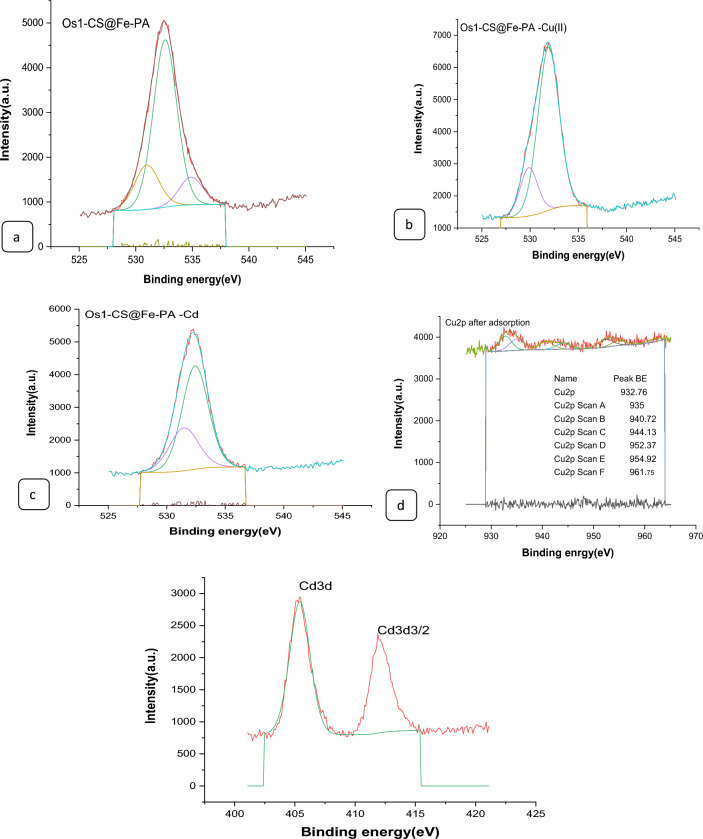
XPS spectra of CS@Fe-PA composite of O1s (**a**) before and after Cu^2+^(**b**); (**c**) Cd^2+^ uptake; (**a**) Cu2p after adsorption Cu^2+^; (**b**) and after adsorption Cd3d.

**Figure 8:**
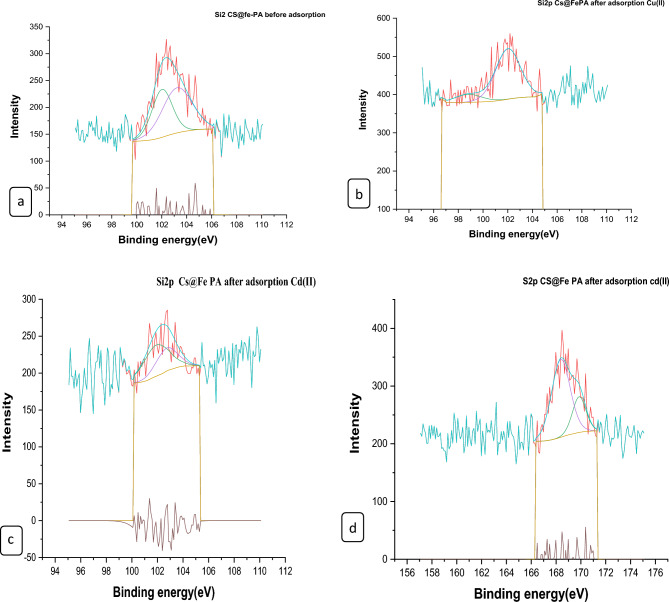
XPS spectra of (**a**)Si2p of CS@Fe-PA before and after Cu^2+^(**b**)/(**c**)Cd^2+^ uptake: and (**d**) Cd 3d 3d3/2 .

**Figure 9:**
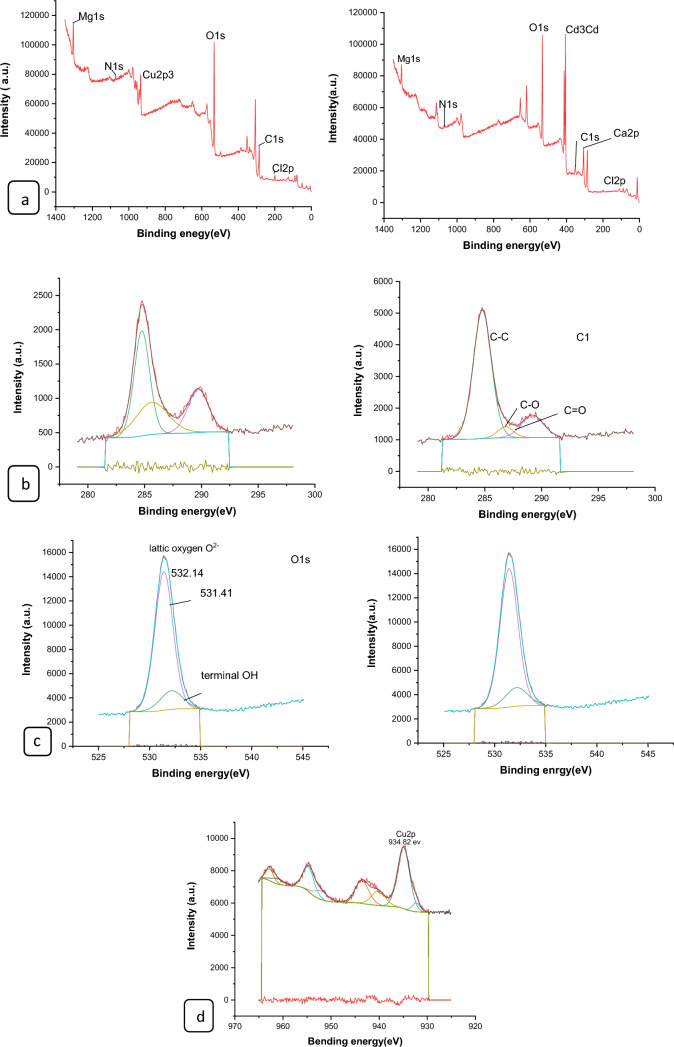
XPS characterization of MgO@Pp before and after Cu2 + adsorption. (**a**) XPS survey spectra along with the spectra of (**b**) C1s, (**c**) O1s, and, (**d**) Cu2p.

**Figure 10:**
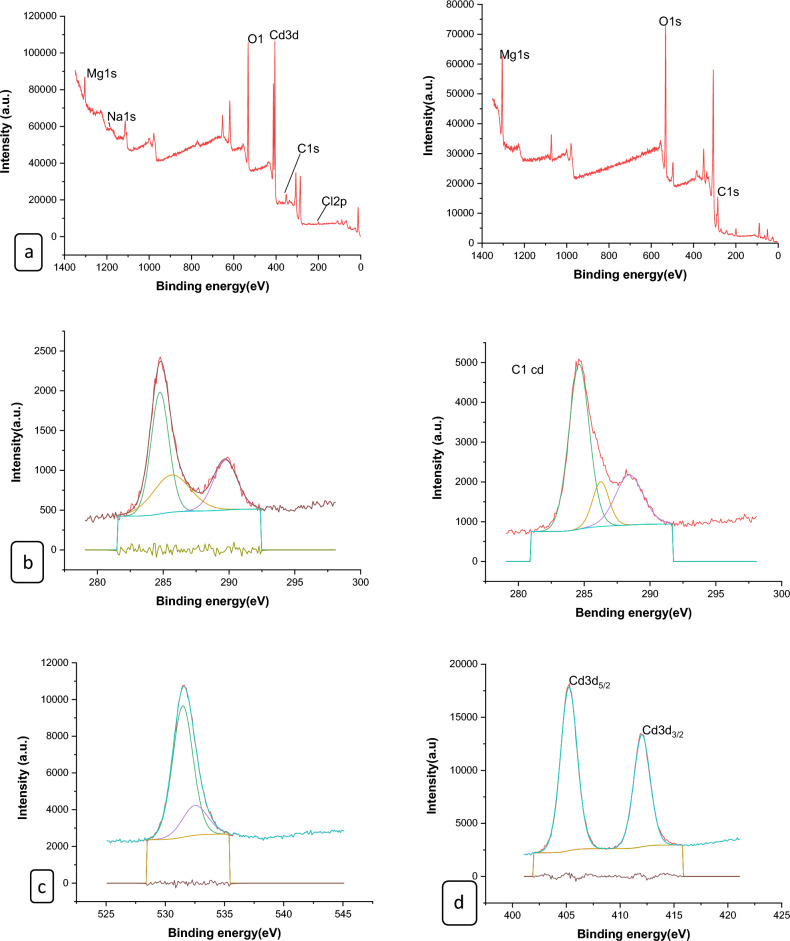
XPS characterization of MgO@Pp before and after Cd2 + adsorption. (**a**) XPS survey spectra along with the spectra of (**b**) C1s, (**c**) O1s, and, (**d**) Cd3d.

The surface chemical compositions of the CS@Fe-PA composite were assessed through XPS analysis, as depicted in Fig. 6a; the results indicated that the MgO@Pp composite consisted of C, N, O, Cu, and Cd elements, whereas the CS@Fe-PA composite contained C, N, O, Fe, and Si elements. The C1s spectrum of CS@Fe-PA Fig. 6b exhibited three peaks prior to adsorption. The first peak, at a binding energy of 284.77 eV, corresponded to C–C, C–H, and C–N bonds. The second peak, at 286.29 eV, was attributed to the C–O bond. The third peak, appearing at 287.36 eV, indicated the presence of C=O bonds in the amide group and C=N bonds ^47^. Upon comparing the C1s spectra after Cu^2+^ and Cd^2+^ adsorption, a decrease in the binding energy of the C–N, C–C, and C–H bonds (284.67 eV) and the binding energy of the C–O bond (286.26 eV), was observed, which may provide electronic energy for the Cu2+ and Cd2+ reduction^43^. Additionally, a slight increase in C=O and C=N in the bonds (287.86 eV) was noticed. The N1s spectrum (Fig. 6b–e) displayed two peaks at binding energies of 399.29 eV (N–H) and 401.58 eV (N–O)^28^. A noticeable change in chemical bonding was observed after the adsorption of Cu^2+^ and Cd^2+^ ions, evidenced by variations in the peak intensities of the N–H Cu, N–HCd bond and NH_2_ Cu^2+^, NH_2_ Cd^2+^ (398.36, 400.49 and 399.42 eV) and (400.13 and 399.08), respectively. The intensity of the N–O bond significantly increased, indicating chelation between the amino group and ferric ions due to the reduction in electron cloud density of N caused by electron shift in the outer layer, resulting in increased binding energy of N^18,48,49^. The high-resolution XPS spectra of Fe2p exhibited nine peaks at binding energies of 710.67, 725.26, 718.26, 728.72, 721.81, 732.86, 736.51, and 713.71 eV. The doublet at 710.67 and 725.26 eV corresponded to Fe(III) 2p3/2, while the peaks at 713.71 and 728.72 eV represented Fe(III) 2p1/2^50,51^. The XPS analysis revealed the presence of a Fe(III) satellite peak at 718.26, 732.86, and 736.51 eV, confirming the presence of ferric ions near the surface of the CS@Fe-PA composite, as depicted in Fig. 6f^48^. The adsorption mechanism was elucidated by the shift in the position of the O1s peak before and after adsorption, as shown in Fig. 7a–c. Prior to adsorption, the O1s spectra of the CS@Fe-PA composite exhibited three peaks. The binding energy peaks at 532.59, 534.88, and 530.98 eV were assigned to C=O, C–O, and Fe–O, respectively. Following Cu^2+^ adsorption, the deconvoluted O1 s spectra of the CS@Fe-PA composite displayed two peaks decreased at 531.91 and 529.88 eV, corresponding to the groups C=O, Fe–OH and Fe–O, respectively. Following Cd^2+^ adsorption, the deconvoluted O1 s spectrum of the CS@Fe-PA composite also exhibited two peaks at 532.43 and 531.47 eV, attributed to the groups C=O and Fe–OH. Performing a split-peak fit to the Cu2p peak in the support material, seven refined peaks were observed at 932.76, 935, 940.72, 944.13, 952.37, 954.92, and 961.75 eV. The dominant peak at 932.59 eV and 954.92 corresponded to Cu2p and Cu2p1/2, respectively, indicating the presence of the divalent form Cu2p (OH), suggesting adsorption of Cu^2+^ on the surface through coordination bonds with the lone pair of electrons in the N atom of the moiety^52^. Regarding the support material, the Cd3d peak could be refined into two peaks at 405.41 eV and 412.14 eV. The peak at 405.9 eV indicated the electron binding energy of the divalent form Cd3/2^53^, suggesting that Cd(II) formed coordination bonds with lone pairs of electrons in O or N in chitosan hydrogels. Cd^2+^ subsequently trapped these bonds. The new peak at 412.4 eV corresponded to the characteristic peak of metallic Cd3d3/2, indicating the formation of chelates between two or more active functional groups in Cd^2+^ and chitosan hydrogels^54^. The binding energy difference of approximately 7eV between the Cd3d and Cd3d(OH) peaks in the pure CS@Fe-PA composite further confirmed the +2 oxidation state of Cd^25^, as shown in Fig. 7d,e, and f. In the CS@Fe-PA composite, the Si2p spectra exhibited two SiOx signal peaks at 102.02 and 103.24 eV. However, after the adsorption of Cd^2+^ and Cu^2+^ ions, the interaction between Si and these metal ions caused a shift in the peak to higher binding energy, resulting in peaks located at 98.81, 102.02, 102.08, and 102.68 eV, as shown in Fig. 8a–c. The peak of S2p peak appeared after the adsorption of the Cd^2+^ ions^36^. This result showed that the composite films successfully adsorbed Cd^2+^ ion. The peak of S2p in Fig. 8d represents the two combination modes of Fe–S–O under 169.87 eV and 168.38 (–SO3^–^, –SO2^–^) functional groups^55^. This indicated that there was an ion exchange between the negative group (–SO3^–^) on the surface of the adsorbent and the Cd2+ ion. The XPS measurements of MgO@Pp before and after adsorbing Cu^2+^ and Cd^2+^. In the survey spectra of MgO@Pp shown in Fig. 9a,b, signals of Ca, Mg, and O elements were observed, and new signals of Cu^2+^ and Cd^2+^ elements appeared after adsorption, providing further evidence of effective Cu^2+^ and Cd^2+^ adsorption onto MgO@Pp. The high-resolution O1 s spectrum, depicted in Fig. 9c,d displayed two deconvoluted peaks at 531.48 eV and 532.52 eV. Which is assigned to O2– of MgO@Pp O or CuO and the absorbed water, respectively. After the adsorption of Cu^2+^, the two peaks shifted from 531.48 eV (O–H) and 532.51 eV (C–O–C) to 532.14 eV (O–H) and 531.5 eV (C–O–C), respectively^56,57^. The XPS results indicate that the surface of MgO@Pp presents massive hydroxyl groups, which are responsible for the Cu^2+^ and Cd^2+^ adsorption. Analyzing the C1s spectrum of MgO@Pp as shown in Fig. 9e,f, three fitting peaks were observed. The first peak at a binding energy of 284.75 eV was attributed to C–C, C–H, and C–N bonds. The second peak at 285.64 eV corresponded to the C=O bond in the amide group and the C=N bond^47^. The third peak at 289.30 eV indicated the M-CO3 bond^58^. Upon comparing the C1s spectra before and after adsorption, a slight increase in the binding energy of each chemical bond was observed, with values of 284.78 eV (C–N, C–C) and a slight decrease in C–H bond (285.4 eV for C–O). Notably, a significant increase in peak intensity was observed for the C=O/C=N bond, indicating the chelation between the amino group and the metal ion. Moreover, a considerable change in the intensity of the M-CO3 peak was observed after C1s adsorption, attributed to the adsorption interaction between the small particles of nanomaterials with a large specific surface area and active sites on the surface with metal ions. XPS spectrum of Cu2p, nine strong satellite peaks were observed at 934.82, 952.0, 940.37, 954.62, 962.76, 961.15, and 943.42 eV, indicating the presence of Cu^2+^ in the MgO@Pp composite^59^. The gap between Cu2p3/2 and Cu2p1/2 was approximately 29 eV, consistent with the CuO standard spectrum, as shown in Fig. 9g ^60^ The presence of Cu shakeup satellite peaks at 943.5 eV and 961.2 eV could be attributed to the Cu2O phase, which is in line with a similar finding reported by^41^. The XPS survey spectrum of Cd(II)- MgO@Pp in Fig10a,b shows peaks at ca. 531.34 eV for O1s and ca. 284.61 eV for C1s; two characteristic peaks of Cd(II) were observed in the Cd3d region at binding energies of 405.24 and 412.4 eV, corresponding to Cd3d5/2 and Cd3d3/2, respectively. The binding energy difference between the Cd3d5/2 and Cd3d3/2 peaks is approximately 7 eV for the MgO@Pp composite, further confirming the +2 oxidation state of Cd^28^. The C1s spectrum (Fig. 10b) of MgO@Pp exhibited three fitting peaks. The first component of binding energy at 284.75 eV was assigned to the C–C, C–H, and C–N bonds. The second component at 285.64 eV was attributed to the C–O bond. The last peak at 289.72 eV corresponded to the C=O bond. By comparing the spectra of C1s after adsorption, a slight decrease in the binding energy of each chemical bond, that is, 284.61 eV (C–N, C–C, and C–H), and 288.41 eV (O–C=O ) was observed. However, a considerable increase in the peak intensity was observed at the C=O bond because of the increase in C=O bonds, which indicated the chelation between the MgO@Pp and metal ion^17^.

### Effects of initial pH on Cu^2+^ and Cd^2+^ adsorption

**Figure 11:**
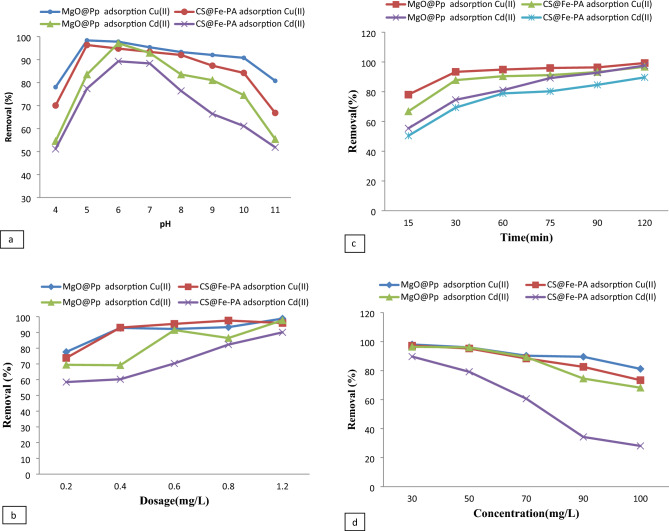
effect of pH (**a**), dose (**b**), contact time(**c**), and initial concentration (**d**), of Cu^2+^ and Cd^2+^ uptake onto CS@Fe-PA and MgO@Pp.

The pH of the solution plays a crucial role in the removal of metal ions from water, as it impacts the surface charge of the adsorbent and consequently alters the adsorption capacity. In this study, the impact of pH on the adsorption efficiency of CS@Fe-PA and MgO@Pp composites for Cu^2+^ and Cd^2+^ ions was evaluated by preparing a solution containing Cu^2+^ and Cd^2+^ ions (50 mg/L) and adding 200 mg of nano-adsorbents, while adjusting the solution's pH within the range of 4–9. The removal percentages of metal ions were plotted against pH in Fig. 11a. The displayed result shows the maximum uptake for Cu^2+^ (98.3%) and (96.3%) at pH 5, and for Cd^2+^ (97.1%) and (89.2%) at pH6 for CS@Fe-PA and MgO impregnated pomegranate peels composites, respectively, were obtained. The pH of the zero point of charge (pHzpc) was found to be 7.5 and 7.1 for CS@Fe-PA and MgO@Pp composites, respectively. The surfaces of CS@Fe-PA and MgO@Pp composite sorbents are negatively charged when pH > pHzpc, which favors metal ions adsorption. The adsorption mechanisms observed on the adsorbents were primarily attributed to the ionic interactions between metal ions and the functional groups present in the nanocomposites^61^. At low acidic pH values, the adsorption efficiency was relatively poor due to the protonation of specific functional groups on the nanocomposite surfaces, leading to electrostatic repulsion between the protonated groups and the metal ions, thereby reducing metal ion adsorption on the CS@Fe-PA and MgO@Pp composites. A lower pH leads to an abundance of hydronium ions (H_3_O^+^) in the solution that causes competition between hydronium ions and Cu^2+^or Cd^2+^ ions (copper and cadmium ions are present as Cu^2+^, Cu(OH)+, Cd^2+^ and Cd(OH)+ at lower pH) for adsorption onto nanoparticles thereby lowering the overall adsorption efficiency of these metal ions at lower pH^8^.

On the contrary, the highest level of adsorption was observed at pH 6. This can be attributed to the deprotonation of functional groups, such as carboxylic acid, hydroxyls, and phenol, on the nanoparticle surface. This deprotonation results in the creation of harmful sites on the nanoparticles, leading to an electrostatic attraction between these negatively charged adsorption sites and the positively charged Cu^2+^ and Cd^2+^ ions. Consequently, this electrostatic interaction enhances the adsorption of these ions^62^. Similar findings have been reported in other studies examining the adsorption of Cu^2+^ onto various adsorbents. For instance, the synthetic *Saccharomyces sp* on the surface of CS/magnetic nanoparticles had an adsorption efficiency of 95.1% at pH 4.55^63^. In comparison, chitosan-modified magnetic kelp *Padina pavonica* had an efficiency of 90.4% at pH5^21^. Zeolite-13X-immobilized *Chlorella*-Alginate showed maximum adsorption of Cu^2+^ (65.6%) at pH5, and magnetically activated carbon nanocomposite (MAC) had an efficiency of 90.2% for Cd^2+^ at pH6^64,65^. However, compared to these studies, CS@Fe-PA and MgO-impregnated pomegranate peels performed better in the treatment of Cu^2+^ and Cd^2+^ in solution. It can be assumed that it was the presence of different functional groups on the surface of activated carbon that led to the increased binding of more ions, as suggested by^49^. Therefore, CS@Fe-PA and MgO impregnated pomegranate peels have a good potential to be utilized as effective adsorbents for the treatment of heavy metals.

### Effects of adsorbent dosages on Cu^2+^ and Cd^2+^ adsorption

The adsorption of Cu^2+^ and Cd^2+^ ions from the aqueous solution is dependent on the adsorbent dosage. This study used a range of doses, from 0.2 to 1.2 g/L, to evaluate this impact. The dosage of the adsorbent determines the availability of potential active adsorption sites and, subsequently, impacts the uptake of metal ions. The adsorbent dosage was investigated to evaluate the adsorption capacity of CS@Fe-PA and MgO@Pp composites for the removal of Cu^2+^ and Cd^2+^ from aqueous solutions. The adsorption efficiency of Cu^2+^ and Cd^2+^ ions by the adsorbents at various dosages is presented in Fig. 11b. The adsorption capacities of both CS@Fe-PA and MgO@Pp composites showed a slight increase with rising dosage, as presented in Fig. 11b. At dosages of 0.2 g/L, the CS@Fe-PA composite exhibited removal efficiencies of 73.4% and 58.4% for Cu^2+^ and Cd^2+^, respectively. At 1.2 g/L, the efficiencies increased to 96.06% and 89.6%. The efficiency of MgO@Pp showed an increase in Cu^2+^ from 77.6% to 98.8% as the dosages increased from 0.2 to 1.2 g/L. In contrast, the removal efficiency for Cd^2+^ ions reached 69.4% at a dosage of 0.2 g/L and increased to 97.07% at a dosage of 1.2 g/L. The incorporation of iron into the adsorbent brings about changes in physicochemical interactions, including aggregation, ion exchange, redox reactions, and hydroxylation, resulting in the continuous precipitation of copper and Cd hydroxides^43^. The inclusion of Fe (III) in the composite improved the chitosan-supported *padina povince* required dosage per the quantity of Cu^2+^ and Cd^2+^ adsorptive. This improvement can be attributed to the creation of negatively charged surface complexes at pH five and the influence of Fe(III) speciation, which promotes the rate of Cd^2+^ adsorption from the test solution^66^. Furthermore, an increase in Cu^2+^ uptake was observed due to the augmentation of active pore spaces on the adsorbent surfaces. Figure 11b illustrates a sharp increase in the Cu^2+^ ion removal trend for both the MgO@Pp and CS@Fe-PA composites within the dosage range of 0.2–1.2 g/L. By comparing the adsorption percentages of the two composites, it is evident that the MgO@Pp composite exhibited higher adsorption rates even at lower quantities of the composite. The enhanced adsorption of the MgO-impregnated pomegranate peel composite can be attributed to its greater surface area and crystallite size when compared to the CS@Fe-PA composite. According to studies conducted by^67^, increasing the adsorbent dose provides more additional internal pore space, facilitating the adsorption of metals and resulting in the effective removal of Cu^2+^, Cd^2+^ and lead. Similarly^7^, reported an increase in the Cd^2+^ removal rate from 81.6 to 99.8% when the sorbent dose of chitosan impregnated with nanoparticles of zero-valent iron was increased from 0.2 to 0.7 g/L^65^. Found that the maximum removal efficiency uptake of Cd^2+^ ions by MAC was 65% at an adsorbent dosage of 0.2 g/L^52^. Reported that at a concentration of 0.5 g/L and a pH of 5, the removal efficiency rate of Cu^2+^ by Fe_2_O_3_ in microalgae was 78.88%^51^. Reported that at a concentration of 0.5 g/L and a pH of 5, the removal efficiency rate of Cu^2+^ by Fe_2_O_3_ in microalgae.

### Influence of contact time on nano composites

In this study, the contact time between the adsorbent and adsorbate was optimized to determine the efficiency of CS@Fe-PA and MgO@Pp composite adsorbents in removing Cu^2+^ and Cd^2+^ ions from aqueous solutions. The contact time was varied within the range of 15 min to 120 minutes, and the results are presented in Fig. 11c. Initially, the adsorption rate increased rapidly within the first 15 minutes (shorter time), resulting in removal efficiencies of 78.04, 55.4, 66.8, and 50.3% for Cu^2+^ and Cd^2+^ in the MgO@Pp and CS@Fe-PA composites, respectively. Subsequently, the adsorption rate slowed down until equilibrium was achieved within 120 minutes (a longer time), leading to removal efficiencies of 98.2, 97.5, 96.7, and 90.09% for Cu^2+^ and Cd^2+^ in the MgO@Pp and CS@Fe-PA composites, respectively. After reaching equilibrium, the sorption system remained relatively constant with increasing contact time, indicating the saturation of biosorption. Therefore, a contact time of 120 min was deemed optimal for subsequent experiments. It was also observed that the adsorption changes in the initial stage were slightly higher in the MgO@Pp composite compared to the CS@Fe-PA composite (Cu^2+^ and Cd^2+^ per 15 minutes of contact time). This can be attributed to the larger surface area of the adsorbent and the availability of numerous vacant active adsorption sites for biosorption. The slower sorption rates observed in the later stages can be attributed to the rest of the sites being difficult to occupy due to repulsive forces between the solute molecules on the solid and bulk phases^68^. Comparable investigations have reported similar findings: a 68.6% adsorption of Cd^2+^ onto graphene oxide/iron(III) oxide nanocomposites within 20 min^61^, as well as a 57% adsorption of Cu^2+^ onto (FeNPs@HC) in the first 30 min^27^. Furthermore, Cu^2+^ exhibited faster adsorption and higher percentage removal compared to Cd^2+^. This difference may be attributed to variations in hydrolysis constants, ionic radius^69^ electrode potential, and solubility of these ions^70^. It is probable that Cu^2+^ ions diffuse more rapidly through the pores of MgO@Pp and are more readily adsorbed compared to Cd^2+^ ions, leading to the quicker and higher removal of Cu^2+^ compared to Cd^2+^ ions in this study. Similar findings have been reported in previous literature^61^ concerning Cd^2+^ adsorption on chitosan-graphene oxide-iron(III) oxide hydroxide, where a maximum adsorption efficiency of 68.7% for Cd^2+^ removal was achieved within 20 min.

### Influence of initial concentration of Cu^2+^ and Cd^2+^ ions

The initial concentration of metal ions has a significant impact on the effectiveness of metal uptake. In this study, initial concentrations ranging from 30 to 100 mg/L were chosen to compare the removal of Cu^2+^ and Cd^2+^. The impact of the initial metal ion concentration on the adsorption activity of the MgO-impregnated pomegranate peels and CS@Fe-PA composites is depicted in Fig. 11d. As the concentration of metal ions increased, the removal efficiency of Cu^2+^ and Cd^2+^ by the adsorbents decreased. The CS@Fe-PA composite showed less sensitivity to increasing initial adsorbate concentrations compared to the MgO@Pp composite. The removal efficiency of Cu^2+^ and Cd^2+^ by the MgO@Pp composite decreased from 98.02 to 81.2% and 96.4 to 68.2%, respectively, when the initial concentrations were increased from 30 to 100 ppm. On the other hand, the CS@Fe-PA composite achieved maximum removal efficiencies of 97.2 to 73.4.9% for Cu^2+^ and 89.7 to 32.08 % for Cd^2+^ when the concentration increased from 30 to 100 ppm. At lower concentrations, specific sites on the adsorbents are utilized for metal adsorption. However, at higher metal concentrations, these sites may become saturated, and the exchange sites can be occupied. This phenomenon has been observed in previous studies^71^. The adsorbent's capacity to hold adsorbate ions remains unchanged at a fixed adsorbent quantity, but with increasing adsorbate concentration, the number of adsorbate ions to be accommodated increases. Consequently, adsorption becomes more rapid at higher initial adsorbate concentrations^72^. The findings are congruent with previously published studies of Cd^2+^ adsorption^73^ confirmed that the titanium-modified ultrasonic *Padina pavonica* removed 59% of cadmium from the aqueous solution. In comparison to the research of^74^, it was reported that the maximum removal efficiencies of Cd^2+^ ions by the corn cobs supporting nano-zero valent iron were 89.0% and 60.1%, respectively, ranging from 25 to 200 mg/L, while the maximum removal efficiencies of Cu^2+^ ions by the macro-algae adsorbent with (CS) and ferric oxide were 48–98%, ranging from 50 to 250 mg/L^21,48^. Found that the maximum removal efficiency of Cu^2+^ ions by the ZnO modified Padina pavonica was 61.02%. These findings are consistent with previous research on Cd^2+^ adsorption^73^. Demonstrated that titanium-modified ultrasonic *Padina pavonica* removed 59% of cadmium from aqueous solutions. Similarly^74^, reported maximum removal efficiencies of Cd^2+^ ions ranging from 89.0% to 60.1% using corn cobs supported by nano-zero valent iron at concentrations ranging from 27 to 200 mg/L. For Cu^2+^ ions^21^, achieved removal efficiencies of 48% to 98% using a macro-algae adsorbent with CS/Fe_2_O_3_ at concentrations ranging from 50 to 250 mg/L. Additionally^75^, reported a maximum removal efficiency of 61.02% for Cu^2+^ ions using ZnO-modified *Padina pavonica*.

### Kinetic study

In order to study the adsorption kinetics mechanism of Cu^2+^ and Cd^2+^ions onto over-surface CS@Fe-PA and MgO@Pp composites. Four kinetic models were utilized to evaluate the adsorption kinetics, including the pseudo-first-order kinetic model (Eq. 3)^41^, pseudo-second-order kinetic model (PSO) Eq. (4) ^12^, Elovich model Eq. (5), and intraparticle diffusion Eq. (6)^76^. These models were used to assess the suitability of each model based on the goodness of fit of the straight line (R^2^) and the agreement between experimental and predicted values of qe. The Weber-Morris Eq. (6) is used to test whether the metal ion species can penetrate the interior sites of the adsorbent particles. The formula for the equation is as follows: Kp is the intraparticle diffusion rate constant, 't' denotes the agitation time in minutes, 'α' denotes the initial adsorption rate (mg/g min), and 'β' represents the desorption constant grams per milligram during any given experiment. The parameter C (mg/g) represents the influence of the boundary layer effect, while Ki (mg g^−1^ min^−1/2^) signifies the rate constant within the intra-particle diffusion model, as described by^77^. The results obtained from fitting data using the pseudo-1st-order, PSO, and Elovich models for CS@Fe-PA and MgO@Pp composites are presented in Table 1. For the pseudo-1st-order model using Eq. (3), values of K1 (0.032), qe (41.3), and R^2^ (0.892) were obtained for Cu^2+^, while K1 (0.025), qe (26.5), and R^2^ (0.874) were obtained for Cd^2+^ in the CS@Fe-PA composite. In contrast, for the MgO@Pp composite, K1 (0.018), qe (48.1), and R^2^ (0.926) were obtained for Cu^2+^, and K1 (0.054), qe (26.2), and R^2^ (0.844) were obtained for Cd^2+^, as depicted in Fig. S1a–d(supplementary materials). These values were calculated by analyzing the linear form of the pseudo-*1st order* kinetic model using Eq. (4). The PSO kinetic is based on mass transfer dynamics. Based on the nonlinear form of the pseudo-2nd-order kinetic model using Eq. (4) and the data presented in Table 1, Cu^2+^ and Cd^2+^ ions in CS@Fe-PA exhibited K2 values of 0.0032 and 0.022, qe values of 45.4 and 47.6 mg/g, and R^2^ values of 0.989, and 0.978, respectively. In comparison, Cu^2+^ and Cd^2+^ ions in MgO@Pp demonstrated K2 values of 0.0038 and 0.014 and qe values of 47.6 and 50 mg/g. The observed R^2^ values for Cu^2+^ and Cd^2+^ were 0.99, 0.990, 0.98, and 0.97, respectively, for CS@Fe-PA and MgO@Pp composites as depicted in Fig. S2a–d (supplementary materials).

**Table 1:** Kinetic parameters for the adsorption Cu^2+^ and Cd^2+^ions onto CS@Fe-PA and MgO@Pp composites.

Model	Kinetic parameter	CS@Fe-PA		MgO@Pp	
		Cu^2+^	Cd^2+^	Cu^2+^	Cd^2+^
Pseudo first- order	qe1 cal.(mg/g)	41.3	26.5	43.4	26.2
	qe2 exp. (mg/g)	48.6	49.7	48.1	49.9
	K_1_ (min^-1^)	0.032	0.025	0.018	0.054
	R^2^	0.89	0.874	0.926	0.844
Pseudo second-order	qe2 exp. (mg/g)	49.7	49.9	49.6	49.1
	qe2 cal. (mg/g)	45.4	47.6	47.6	50
	K^2^ (min^-1^)	0.0032	0.022	0.003	0.014
	R^2^	0.98	0.97	0.99	0.99
Elovich	α	0.69	1.7	0.48	0.44
	β	3.6	3.8	3.4	3.5
	R^2^	0.973	0.964	0.981	0.976
Intraparticle diffusion	Kpi1(mg/g min^1/2^)	0.374	0.05	0.297	0.237
	Kpi2(mg/g min^1/2^)	0.08	0.02	0.126	0.106
	Kpi3(mg/g min^1/2^)	0.005	0.03	0.041	0.033
	Ci-1	32.3	46.4	46.4	39.6
	Ci-2	36.8	47.62	47.02	41.6
	Ci-3	41.7	48.08	48.08	44.7
	R^2^	0.97	0.98	0.99	0.99
	R^2^	0.87	0.94	0.97	0.92
	R^2^	0.67	0.68	0.66	0.74

According to the k2 values in Table 1, it was deduced that MgO@Pp adsorbed Cu^2+^ and Cd^2+^ faster than CS@Fe-PA. However, in terms of adsorption capacity, MgO@Pp exhibited higher values (qe = 47.6 and 50 mg/g, respectively), whereas the corresponding values for CS@Fe-PA were 45.5 and 47.6 mg/g, respectively. It was found that the highest initial adsorption rate (h) was achieved when utilizing MgO@Pp, as indicated in Table 1. These values indicate that the adsorption of metal ions in CS@Fe-PA and MgO@Pp composites followed the pseudo-2nd order kinetic model. Considering these findings, it can be deduced that the optimal percentage of MgO in the composite with pomegranate peels contributes to enhanced pollutant adsorption. Therefore, the MgO@Pp composite proves to be a superior material for the adsorption of metal ions.

### Elovich model

The Elovich equation is a highly regarded model for analyzing activated chemisorptions. It is defined by equation (5). By plotting qe against ln(t), graphs can be generated to calculate α, β, and the correlation coefficient (R^2^) using Table 1. According to^78^, the chemical reaction of Cu^2+^ and Cd^2+^ with the adsorbent surface is the rate-controlling step of the Elovich model, and the removal process involves multilayer adsorption. The results reveal that the initial Cu^2+^ and Cd^2+^ adsorption rates onto CS@Fe-PA and MgO@Pp were studied. The Elovich values (α) of Cu^2+^ and Cd^2+^ were determined to be 0.69, 1.7, 0.48, and 0.44 mg min^-1^, respectively, for MgO@Pp and CS@Fe-PA composites. The values of Cu^2+^ and Cd^2+^ of β, which denotes the number of adsorption sites, were 3.6, 3.8, 3.4, and 3.5 for MgO@Pp and CS@Fe-PA, respectively. The adsorption of Cu^2+^ and Cd^2+^ onto MgO@Pp and CS@Fe-PA follows the Elovich equation because of the high R^2^ values. Fig. S3a–d (Supplementary Materials) and Table 1 indicate that the Elovich equation can describe the adsorption of Cu^2+^ and Cd^2+^ on MgO@Pp and CS@Fe-PA. These findings suggest the heterogeneity of adsorbent surfaces and support the prediction of a chemisorption process as indicated by the PSO model^79^.

### Intra-particle diffusion model

The transfer of solutes in the solid-liquid adsorption process is commonly analyzed using an intra-particle diffusion model, as depicted in equation (6). By plotting qt against t1/2, the slope and intercept of the linear graph can be used to determine the Ki and C values from equation (6), as shown in Table 1. The Ri value is divided into four zones: weakly initial adsorption (zone 1) for 1 > Ri > 0.9, intermediately initial adsorption (zone 2) for 0.9 > Ri > 0.5, strongly initial adsorption (zone 3) for 0.5 > Ri > 0.1, and approaching completely initial adsorption (zone 4) for Ri < 0.1. Table 1 presents the collected and estimated intraparticle diffusion constants for the adsorption of Cu^2+^ and Cd^2+^ onto MgO@Pp and CS@Fe-PA composites. The first step exhibited excellent linearization (R^2^ = 0.99, 0.99, 0.97 and 0.98) with the first intraparticle rate constant (Kid1) of 0.297, 0.237, 0.374 and 0.05 mg/ (g/min^0.5), respectively, for indicating a rapid process. This initial step could be attributed to film diffusion, where Cu^2+^ and Cd^2+^ ions molecules diffuse from the solution onto the external surfaces of the MgO@Pp and CS@Fe-PA composites, possibly forming a hydrodynamic boundary layer. In contrast, the subsequent steps demonstrated a decline in the second intraparticle rate constant (Kid2) of 0.126, 0.106, 0.08 and 0.026 mg/(g/min^0.5) and the third intraparticle rate constant (Kid3) of 0.041, 0.033, 0.005 and 0.03 mg/(g/min^0.5), along with lower R^2^ values (R^2^ = 0.66,0.75,0.67 and 0.68) from the Webber–Morris equation. The initial sharper part corresponds to the fast diffusion of Cu^2+^ and Cd^2+^ ions from the solution bulk to the external surface of CS@Fe-PA and MgO@Pp composites, or the boundary layer. The second stage of the adsorption process encompasses film diffusion and intra-particle diffusion mechanisms, where solutes gradually migrate from the boundary layer to the surface of the adsorbent. Subsequently, they attach themselves to the active sites within the pores. The last linear segments represent the equilibrium period, where the intraparticle diffusion rates of cations (kint, 3) significantly decrease, and equilibrium is gradually attained^80^. The recorded C values for the adsorption of Cu^2+^ and Cd^2+^ ions on CS@Fe-PA were 32.3, 36.8, and 41.7 mg/g, and 46.4, 47.02, and 48.08 mg/g, respectively. Similarly, MgO@Pp exhibited values of 46.4, 47.02, and 48.08 mg/g for Cu^2+^ ions and 39.6, 41.6, and 44.6 mg/g for Cd^2+^ ions. These values provide insights into the thickness of the boundary layer. It is noted that the influence of the boundary layer becomes more significant as the intercept increases^81^**.** Both Ki and C values of the MgO@Pp composite were the highest, indicating that the diffusion rate in this composite was faster, and surface sorption contributed more than in the case of CS@Fe-PA^18^.The observed phenomenon can be attributed to the relatively smaller pore size of the MgO-impregnated pomegranate peels composite, allowing for easier penetration of the metal ion molecules. Moreover, the initial adsorption of metal ions over MgO@Pp and CS@Fe-PA composite adsorbents belonged to zone 3 with 0.5 > Ri > 0. 1, initial solid adsorption, and zone 4, Ri < 0.1, complete initial adsorption. These classifications were determined based on the calculated Ri values^82^.

### Adsorption isotherms

The Langmuir and Freundlich model can be expressed in its linear form, along with the separation factor RL (Eqs. 7 and 9). The composite's adsorption capacity is denoted as qe, expressing the quantity of metal in milligrams per gram of dry adsorbent. Meanwhile, Ce, measured in milligrams per litre, represents the concentration of metal ions following the treatment. Qmax, expressed in milligrams per gram, corresponds to the Langmuir constant. KL, measured in liter per milligram, is the constant associated with the free energy or net enthalpy of adsorption. KF represents the Freundlich constant, which is measured in milligrams per gram and is related to the adsorption capacity. The Freundlich exponent, denoted as n and dimensionless, serves as an empirical parameter indicating the intensity of adsorption. For favorable adsorption, the value of n should be within the range of 1 < n < 10. Co represents the initial amount of adsorbate, measured in milligrams per liter. The RL parameter provides a more dependable indication of adsorption and can take on four potential values. By computing the slope and intercept from the Langmuir plot of Ce versus Ce/qe in Fig. S4a–d (supplementary materials), the Langmuir constants Qo and b were determined. The Langmuir models disclosed that the maximum adsorption capacities (Qm) of the MgO@Pp composite for Cu^2+^ and Cd^2+^ were 333.3 and 200 mg/g, respectively. In contrast, the Q_m_ values for the CS@Fe-PA composite was 250 and 142.8 mg/g for Cu^2+^ and Cd^2+^, respectively. The Qm of the MgO@Pp composite to Cu^2+^ and Cd^2+^ was greater than that of the CS@Fe-PA composite. The Langmuir R^2^ values were very high, being almost 0.971 and 0.944 for Cu^2+^ and Cd^2+^ by the MgO@Pp as compared to the CS@Fe-PA composites (0.95 for Cu^2+^ and 0.92 for Cd^2+^). The RL values were calculated for the nanoparticles studied using Eq. (8), which takes into account b (L mg^−1^) and Ci (mg L^−1^). For the Cu^2+^ and Cd^2+^ ions in the present system, the RL values ranged from 0.1 to 0.09, and 0.4 to 0.3, respectively, for the MgO@Pp and CS@Fe-PA composites, indicating a favourable adsorption process Table 2. The linear form of the Freundlich adsorption model is represented by Eq. (9). The determination of the Freundlich constants Kf and n involved analyzing the slope and intercept of the plot depicting ln qe versus ln C e, as presented in Fig. S5(supplementary materials). The adsorption intensity (n) values were observed to be greater than 1, confirming the favorable adsorption of the two metal ions investigated using CS@Fe-PA and MgO@Pp composites, as demonstrated in Table 2. Furthermore, the values of Freundlich constants n for Cu^2+^ (1.6) and Cd^2+^ 2.4) for MgO@Pp, as well as constants n for Cu^2+^ (1.4) and Cd^2+^ (1.2), are greater than 1. The determination coefficient values (R2) obtained from the Freundlich model for Cu^2+^ and Cd^2+^ using the MgO@Pp composite were found to be 0.993 and 0.988, respectively. The determination coefficient values (R^2^) obtained from the Freundlich model for Cu^2+^ and Cd^2+^ using the CS@Fe-PA composite was found to be 0.97 and 0.95, respectively. These values were higher compared to the Langmuir model, indicating a better fit of the data to the Freundlich model and confirming the occurrence of multilayer adsorption. From the analysis shown in Fig. S5a–d(supplementary materials), it can be observed that the Freundlich model demonstrated a stronger correlation with the experimental data for both the MgO@Pp and CS@Fe-PA composites in comparison to the Langmuir model. The composites of MgO@Pp and CS@Fe-PA displayed a higher adsorption capacity compared to numerous other absorbents that have been documented in the literature. Temkin isotherm model is mathematically represented by Eq. (10)^83^. The Temkin plot Fig. S6a–d (supplementary materials) and Table 2 provided estimated values for Cu^2+^ and Cd^2+^ of the MgO@Pp composite, with AT = 2.3 L/g, B = 33.18 J/mol, and AT = 2.1 L/g, B = 36.06 J/mol, respectively. For the CS@Fe-PA composite, the estimated values for Cu^2+^ were AT = 2.06 L/g, B = 37.02 J/mol, while those for Cd^2+^ were AT = 2.02 L/g, B = 37.86 J/mol. These values suggest a physical adsorption process, as the Temkin isotherm model with a high correlation coefficient of 0.986 and 0.983 for Cu^2+^ and Cd^2+^ by MgO@Pp composite, respectively. The model was also applied to the CS@Fe-PA composite, resulting in correlation coefficients of 0.979 and 0.956 for Cu^2+^ and Cd^2+^, respectively. These results indicate that the Freundlich model is a better fit for the experimental data of Cu^2+^ and Cd^2+^ adsorption by CS@Fe-PA and MgO@Pp composites than the other two models.

**Table 2:** Equilibrium isotherm modeling of Cu^2+^ and Cd^2+^ adsorption onto adosorbents CS@Fe-PA and MgO@Pp.

Isotherm	CS@Fe-PA		MgO@Pp	
	Cu^2+^	Cd^2+^	Cu^2+^	Cd^2+^
Langmuir				
qe (mg/g)	250	142.8	333.3	200
R^2^	0.959	0.921	0.971	0.944
Freundlich				
n	1.4	1.2	1.6	2.4
KF (L/mg)	8.03	4.32	32.3	36.4
R^2^	0.978	0.957	0.993	0.988
Tempkin				
B (J/mol)	37.02	37.86	33.1	36.06
A_T_ (L/mg)	2.06	2.02	2.3	2.1
R^2^	0.979	0.956	0.986	0.983
Doubinin Radushkevich				
qm Cal(mg/g)	2.5	0.9	3.6	0.7
β (mol2 kJ^−2^)	0.00036	0.00063	0.003	2.13
Ea (kJmol^−1^)	36.8	28.8	12.9	11.3
R^2^	0.97	0.96	0.98	0.98
Redlich–Peterson				
β	0.055	0.045	0.022	0.016
aR (L/mg)	1.336	1.042	1.955	1.75
KRp (L/g)	2.49	9.7	1.171	1.47
R^2^	0.970	0.977	0.987	0.994

#### Dubinin–Radushkevich isotherm

Equations (11, 12) present the non-linear version of the (D-Rh) isotherm^84^. The purpose of this approach was to differentiate between chemical and physical adsorptions of metal ions based on their mean free energy (E). According to Table 2, the sorption energy β for MgO@Pp was determined to be 0.003 (Cu^2+^) and 2.13 (Cd^2+^), while for CS@Fe-PA, β was reported to be 3.6 × 10^–4^ (Cu^2+^) and 6.3 × 10^–4^ (Cd^2+^). For the MgO@Pp composite, the E values for Cu^2+^ and Cd^2+^ were found to be 12.9 and 11.39, respectively, indicating that the sorption process follows an ion exchange mechanism. In contrast, for the CS@Fe-PA composite, the E values for Cu^2+^ and Cd^2+^ were 36.8 and 28.1, respectively. These positive E values suggest that the sorption process is chemisorption. Furthermore, it can be inferred that a higher solution temperature would promote the sorption process, as stated by^85^. The Redlich-Peterson model can be expressed by Eq. (13), as mentioned by^45^. Table 2 displays the heterogeneity (β) values for Cu^2+^ and Cd^2+^ ions, which fall within the range of 0.022 and 0.016 for MgO@Pp and 0.055 and 0.45 for CS@Fe-PA. Table 2 displays the αR (L/g) values for Cu^2+^ and Cd^2+^ ions, which fall within the range of 1.955 and 1.76 for MgO@Pp composite and 1.336 and 1.042 for CS@Fe-PA composite. The Redlich–Peterson isotherms showed the highest fitness to the adsorption data by generating the highest R^2^ values in the range of Cu^2+^ and Cd^2+^ ions 0.987–0.994 and 0.97–0.977, respectively, for MgO@Pp and CS@Fe-PA composites, as shown in Table 2.

#### Thermodynamic studies

Thermodynamic research is an essential tool for understanding the nature of adsorption processes. By analyzing the standard thermodynamic parameters, researchers can determine the nature of an adsorption process, distinguishing between physical and chemical adsorption. In recent studies, these parameters have been used to investigate the removal of Cu^2+^ and Cd^2+^ ions using chitosan @Fe PA and MgO@Pp composites. The assessment of thermodynamic characteristics such as changes in Gibbs free energy (ΔG), enthalpy (ΔH), and entropy (ΔS) associated with the adsorption process can be accomplished by employing both the rate Eq. (15) and the van't Hoff equation. The rate equation can be expressed as presented in previous works^86,87^, and T denotes the adsorption temperature in Kelvin (K). By plotting ΔG° against temperature, a linear correlation was observed. The slope and intercept of the plot enabled the calculation of ΔS° and ΔH° values. Additionally, plotting the natural logarithm of KL against 1/T yielded a straight line, with the slope and intercept corresponding to ΔH°/R and ΔS°/R, respectively. These values facilitated the computation of enthalpy and entropy changes. The equilibrium constant (K), absolute temperature (T), equilibrium concentration (Ce), and the amount of Cu^2+^ and Cd^2+^ adsorbed on the surface of the composites (qe) were also considered in the calculations, as per the equations presented in Eq. (16).The slopes and intercepts of the plot of ln Kc versus 1/T helped to determine the values of ΔH° and ΔS°, as shown in Fig. S6(supplementary materials). Further, ΔG° was calculated using the corresponding ΔH° and ΔS°. Table 3 provides the thermodynamic parameters for the adsorption of Cu^2+^ and Cd^2+^ on the CS@Fe-PA and MgO@Pp composites at different temperatures. The feasibility and spontaneity of the adsorption process are confirmed by the negative ΔG° values, which are further substantiated by the Langmuir separation factor RL and the Freundlich exponent n. The values of RL were between 0 and 1, and n was greater than 1. Raising the temperature caused a decline in the value of ΔG°, suggesting that an elevated temperature promotes the favorability of the adsorption process. The positive value of enthalpy confirms the endothermic nature of the interaction between the prepared MgO@Pp composite and the external boundary layer of Cu^2+^ and Cd^2+^. With an increase in temperature, the negative values of ΔG° became more negative, suggesting that the adsorption of Cu^2+^ and Cd^2+^on the MgO@Pp composite increased. On the other hand, positive values of ΔG° indicate that the adsorption of Cu^2+^ and Cd^2+^ on the CS@Fe-PA is a non-spontaneous process. For all the metals examined, the fact that the enthalpy value is negative indicates that the adsorption is an exothermic reaction. On the contrary, an increase in temperature results in increased mobility of the adsorbate molecules and a reduction in the solution's viscosity, making the subsequent stages of adsorption more favorable. The consistently observed negative enthalpy values confirm that the adsorption process for all the examined metals is indeed exothermic^17^.

**Table 3:** Thermodynamic parameters of Cu^2+^ and Cd^2+^ adsorption activity of CS@Fe-PA and MgO@Pp composites.

Adsorbent	Temperature, K	(ΔG ), kJ mol^-1^	ΔH, kJ mol^-1^	ΔS, kJ mol-^1^	R^2^
MgO@Pp Cu^2+^	298	− 6.7488471	59.09	22.6	0.992
	303	− 6.8620838			
	308	− 6.9753205			
	318	− 7.3150305			
	323	− 7.7679772			
MgO@Pp Cd^2+^	298	− 5.3292162	41.1	17.8	0.985
	303	− 5.4186333			
	308	− 5.5080503			
	318	− 5.7763016			
	323	− 6.1339698			
CS@Fe-PA Cu^2+^	298	9.04797713	− 0.11	− 30.3	0.980
	303	9.19979077			
	308	9.35160441			
	318	9.80704533			
	323	10.4142999			
CS@Fe-PA Cd^2+^	298	11.8253012	− 0.14998	− 39.6827	0.975
	303	12.0237148			
	308	12.2221284			
	318	12.6189556			
	323	12.8173692			

#### Adsorption mechanism

**Figure 12:**
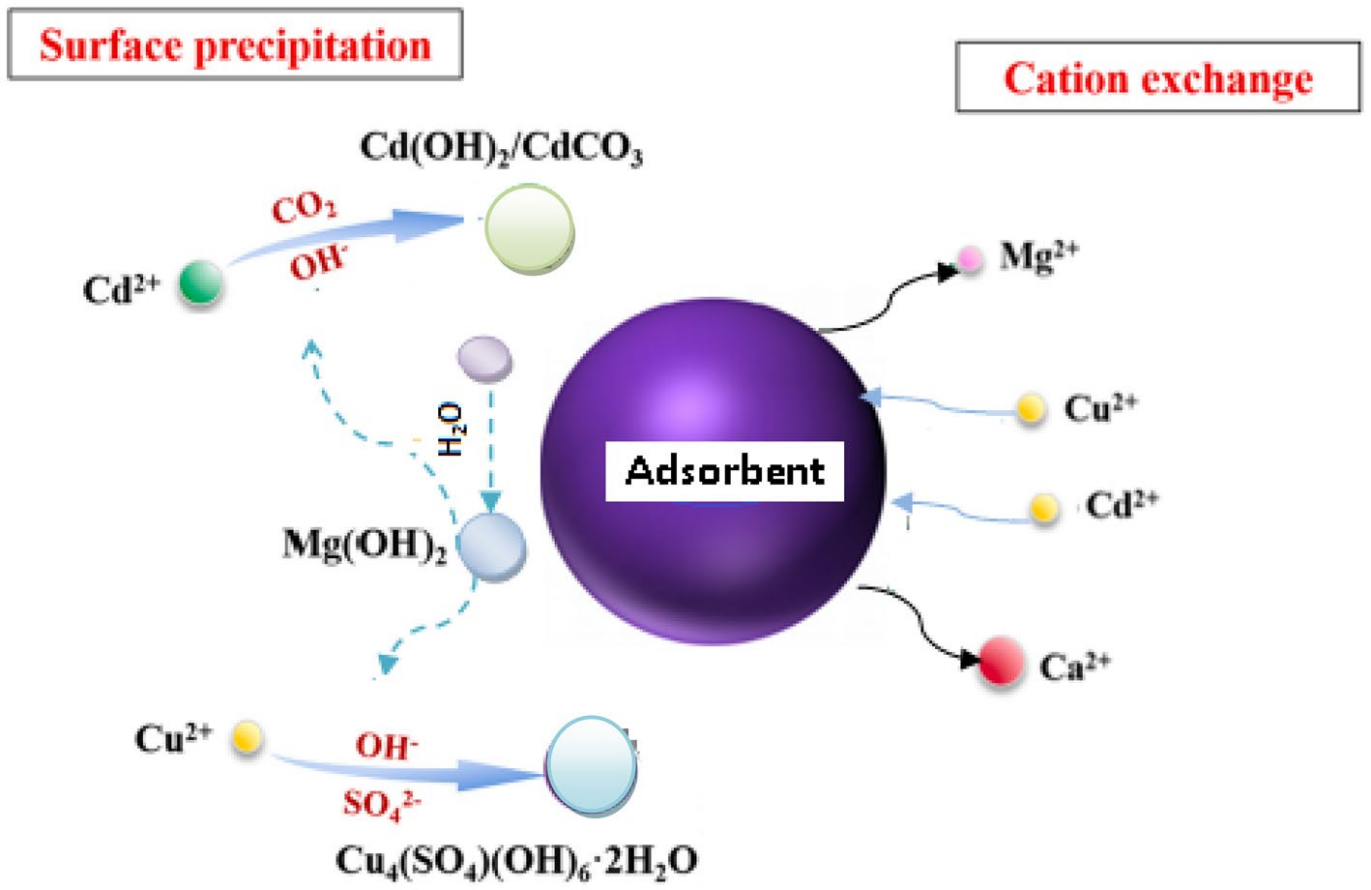
Removal mechanism of Cd^2+^ and Cu^2+^ by CS@Fe-PA and MgO@Pp.

The mechanism of CS@Fe-PA and MgO@Pp on heavy metals usually involves complexation, ion exchange, physical adsorption, precipitation, and the combined effects of these actions^88^. Adsorption mechanisms of Cd^2+^ and Cu^2+^ ions adsorption on the surface of CS@Fe-PA and MgO@Pp can be divided into two categories. The first category refers to physical adsorption (physisorption), in which a one-step reaction mechanism occurs through van der Waals force, which are interaction inversely proportional to the distance between atoms or molecules^21^. The ΔH values were < 40 kJmol1, proving that physical adsorption occurred during the adsorption of Cd^2+^ and Cu^2+^ ions onto CS@Fe-PA and MgO@Pp. The negative zeta potential of CS@Fe-PA and MgO@Pp in the pH range of 5–6 aids in the adsorption of positively charged heavy metal ions through electrostatic attraction. However, the effect of electrostatic attraction on the adsorption of Cd^2+^ and Cu^2+^ ions by CS@Fe-PA and MgO@Pp is relatively small^21^.The pseudo-second-order kinetics and Freundlich adsorption model for heterogeneous adsorbents exhibited excellent correlation with the Cd^2+^ and Cu^2+^ ions adsorption onto CS@Fe-PA and MgO@Pp, proving that the chemisorption process is mainly responsible for the adsorption of Cd^2+^ and Cu^2+^ ions onto CS@Fe-PA and MgO@Pp^76^. The ion-exchange process was attributed to the increase of equilibrium pH. In aqueous solution, the free Cd^2+^ and Cu^2+^ ions were hydrolyzed to produce H + (Eqs. (17) and (18)). During Cd^2+^ and Cu^2+^ ions adsorption process, more Mg^2+^ was released from the surface of MgO@Pp, and Cd^2+^ and Cu^2+^ ions were adsorbed on MgO@Pp through ion-exchange (Eqs. (19) and (20) (Fig. 12). Therefore, less H^+^ was produced from the hydrolysis. The considerable amount of MgO in the MgO@Pp consumed the existing H^+^ (Eq. (21). With the decreased H^+^ produce and the increased H + capture, the equilibrium pH was increased compared to the initial pH during Cd^2+^ and Cu^2+^ ions removal process.

17 $$ {\text{Cu}}^{{{2} + }} + {\text{H}}_{{2}} {\text{O}} \to {\text{Cu }}({\text{OH)}}^{ + + } {\text{H}}^{ + } $$

18 $$ {\text{Cd}}^{{{2} + }} + {\text{H}}_{{2}} {\text{O}} \to {\text{Cd}}\left( {{\text{OH}}} \right) + + {\text{H}}^{ + } $$

19 $$ {\text{MgO}} + {\text{Cu}}^{{{2} + }} \to {\text{CuO}} + {\text{Mg}}^{{{2} + }} $$

20 $$ {\text{MgO}} + {\text{Cd}}^{{{2} + }} \to {\text{CdO}} + {\text{Mg}}^{{{2} + }} $$

21 $$ {\text{MgO}} + {\text{H}} + \to {\text{Mg}}^{{{2} + }} + {\text{H}}_{{2}} {\text{O}} $$

**Figure 13:**
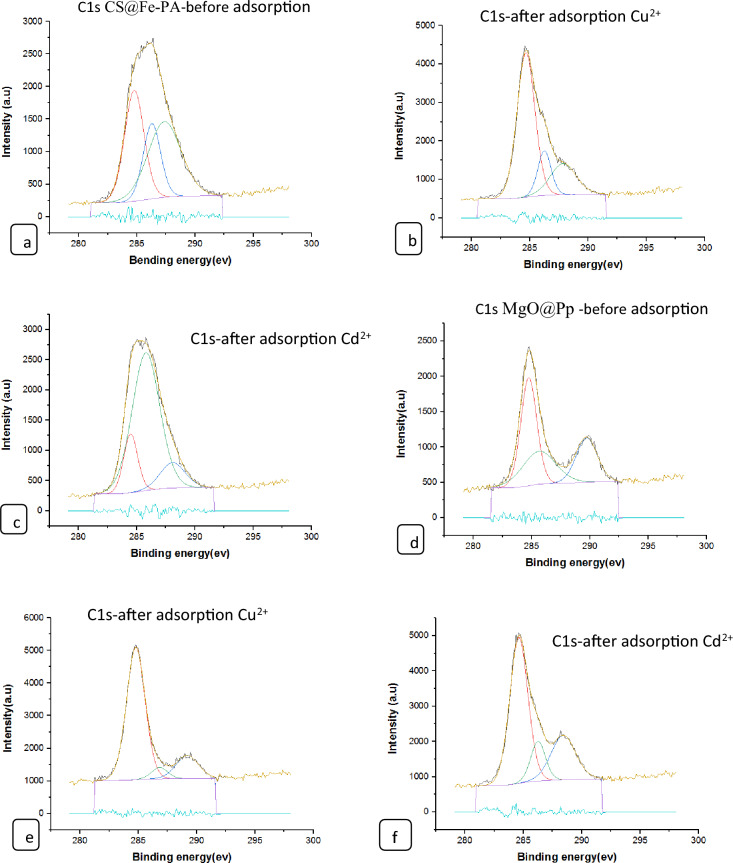

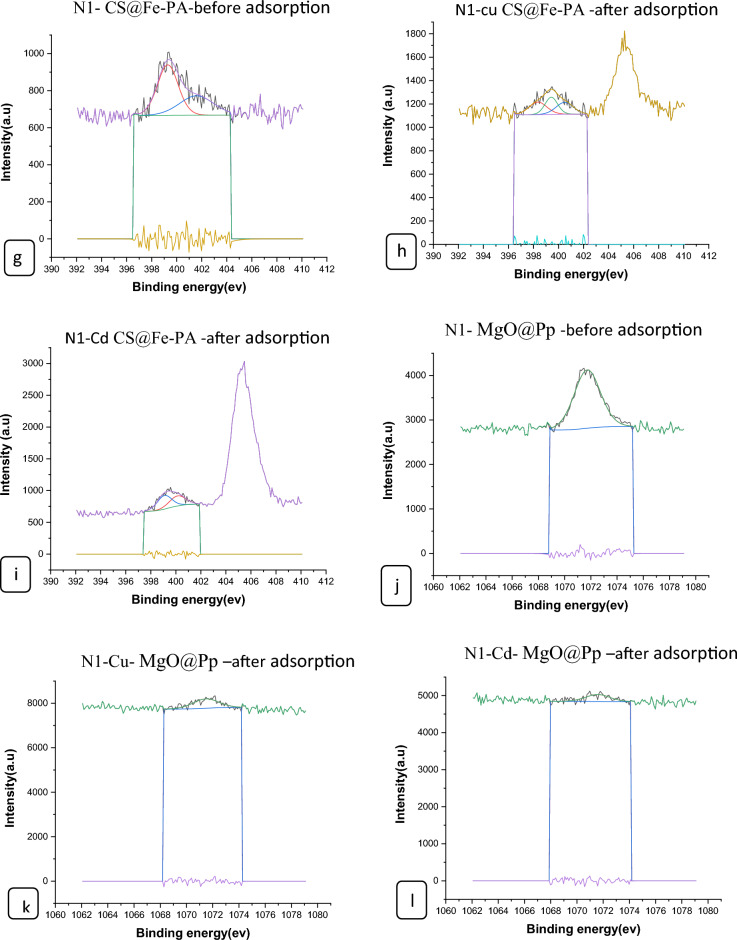

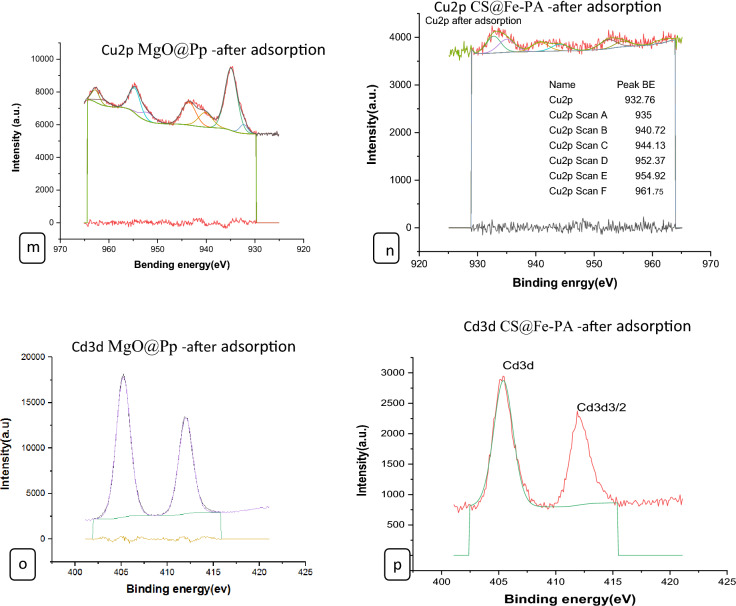
XPS Spectra of C1s (**a**–**f**),Cd3d,Cu2p (**g**–**j**) and N1s (**k**–**p**) before and after MgO@Pp and CS@Fe-PA Cd^2+^ and Cu^2+^ ions adsorption.

The surface complexion depends on the electrostatic attraction between the surface functional groups on the CS@Fe-PA and MgO@Pp and the heavy metal ions in an aqueous solution. Positively or negatively charged groups on the surface, such as hydroxyl, carboxyl, ester, or amine groups, attract oppositely charged ions in the solution, forming the complexions through chemical bonding on the surface. When several different ions or molecules in the solution are adsorbed together on the surface, this process is called co-precipitation^89^. So, both surface complexion and co-precipitation are predominant in the case of CS@Fe-PA and MgO@Pp due to the addition of various functional groups on the surface of the CS@Fe-PA after the introduction of the chitosan layer. The role of surface complexation with these active groups in Cd^2+^ and Cu^2+^ ions sorption was evaluated by characterizing CS@Fe-PA and MgO@Pp with FTIR technique. XPS was also employed to understand the roles of functional groups in the Cd^2+^ and Cu^2+^ ions removal process. The binding energy and the changes of the surface chemical elements before and after adsorption are shown in Fig. 13a–c shows the XPS C1s spectra of CS@Fe-PA and MgO@Pp before and after Cd^2+^ and Cu^2+^ ions adsorption, where the spectra were deconvoluted into three peaks attributable to C–O, C=O, and O–C=O. It can be observed that C–O after adsorption changes significantly after CS@Fe-PA and MgO@Pp adsorbed Cd^2+^ and Cu^2+^ ions, and the binding energy of the corresponding characteristic peaks also shifts significantly. This may be related to the complexation of the Л-electron, which forms the Cd–Л bond between the Cd^2+^ and C=C double bond. It means that metal can form a σ bond with the s orbital of the C=C double bond, and the d orbital of metal can reversely contribute electrons to the Л orbital of the C=C double bond^90^. This result indicates that the C=C double bond may be vital to the adsorption of Cd^2+^. The C1s peak pattern after Cu^2+^ adsorption by CS@Fe-PA and MgO@Pp Fig. 13d–f differs from that before Cu^2+^ adsorption by CS@Fe-PA and MgO@Pp. The characteristic peaks corresponding to carbonyl, carboxyl, and hydroxyl groups change significantly, indicating that carbonyl, carboxyl, and hydroxyl are significant to Cu adsorption^91^.The XPS peaks of N1s elements in CS@Fe-PA and MgO@Pp after adsorption of Cd^2+^ and Cu^2+^ ions are in Fig. 13g–l. In addition, after CS@Fe-PA and MgO@Pp adsorbs Cd^2+^ and Cu^2+^, the characteristic peaks corresponding to these two N-containing functional groups also shift significantly. The obvious shift of these characteristic peaks may be due to complexes between N-containing functional groups and heavy metals. Studies have shown that when the covalent bond formed by N atoms and heavy metals obtains lone pair electrons from N atoms, the electron cloud density on N atoms decreases, thus changing the binding energy of the corresponding characteristic peaks^92^ and shifting them. Figure 13m–p show the XPS Cu2p and Cd3d spectra, respectively. The binding energies of Cu2p and Cd3d were observed after Cd^2+^ and Cu^2+^ ions adsorption, implying that CS@Fe-PA and MgO@Pp ions were successfully adsorbed on the CS@Fe-PA and MgO@Pp. The Cu^2+^/Cu–O were assigned to Cu2p, and Cd^2+^/Cd–O were assigned to Cd3d. At the same time, the Cu–O and Cd–O bonding contributed to the complexation with functional groups^51^, which was consistent with the results of the C1s spectra analysis. O1s spectra were deconvoluted into three peaks at 531.2, 532.1, and 533.3 eV are assigned to the lattice oxygen, bridging hydroxyl, terminal hydroxyl, and the surface adsorption of water, respectively attributable to Mg-O,-OH and C-O (Fig. S7) (supplementary materials). After the adsorption of CS@Fe-PA and MgO@Pp ions, the -OH and C-O groups decreased. This phenomenon is attributed to the ion- exchange between CS@Fe-PA and MgO@Pp and CS@Fe-PA and MgO@Pp ions^93^. To sum up, the mechanism for CS@Fe-PA and MgO@Pp ions adsorption by CS@Fe-PA and MgO@Pp, including precipitation, complexation, and ion-exchange, consequently the ion-exchange is the main mechanism during CS@Fe-PA and MgO@Pp ions adsorption process^88^.

#### Evaluating the adsorption capacity (qmax) of nano composites with diverse adsorbents

Table 4 presents a comprehensive comparison of the adsorption capabilities for Cu^2+^ and Cd^2+^ across various adsorbents documented in existing literature. The findings indicate that the utilization of CS@Fe-PA and MgO@Pp composites, as employed in this research, demonstrates significant potential as an efficient adsorbent for Cu^2+^ and Cd^2+^ ions. Notably, its performance surpasses that of several other adsorbents listed in the table above.

**Table 4:** Comparison of adsorption capacities for metals ions on different adsorbents.

Adsorbents	Dosage (g/L)	q_m_ (mg/g)	References
MgO-biochar	2	835	^1^
MgO-biocha	0.5	233	^2^
Mg-EBC	2	119.6	^3^
MgFe2O4-biochar	0.2	477	^4^
MgO magnetic-biochar	0.05	148	^5^
Chi@Fe3O4	0.5	142	^6^
Chitosan/Hydroxyapatite/nano-Magnetite (Fe3O4)	0.02	15	^7^
Chitosan-coated magnetic nanoparticles	0.15	144	^8^
MgO@Pp	0.2	333	This study
CS@Fe-PA	0.2	200	This study

#### Reusability of the prepared adsorbent composites studies

**Figure 14:**
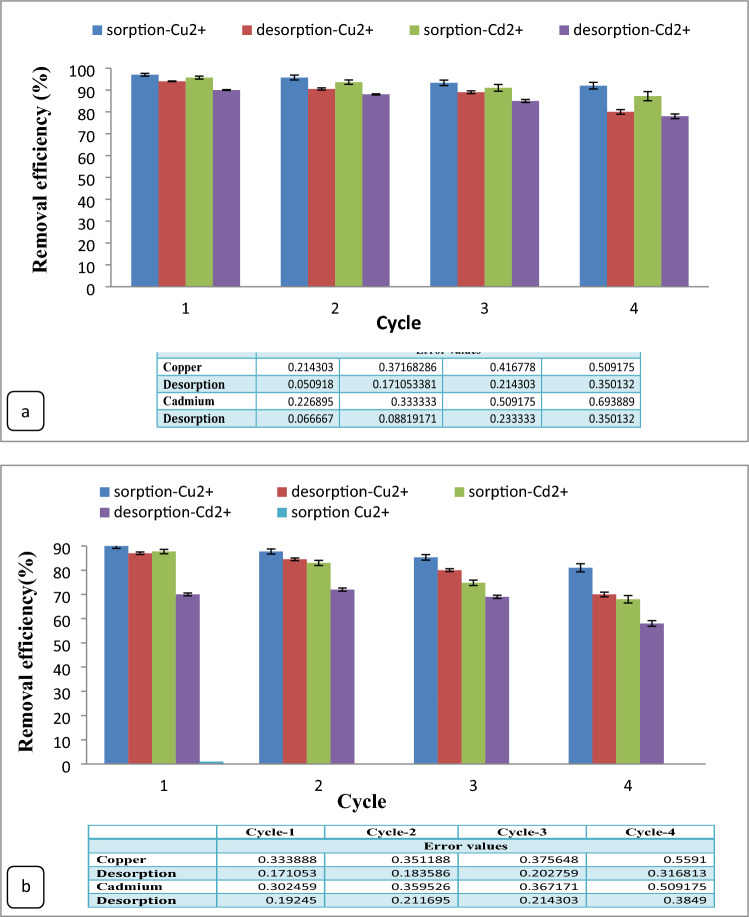
adsorption–desorption cycles of MgO@Pp (**a**) and (**b**) CS@Fe-PA composite for Cu^2+^ and Cd^2+^ ions.

Reusability is an indispensable indicator for evaluating the longevity of an adsorbent. In this study, a 0.2 mol/L HCl solution and deionized water were respectively used to regenerate CS@Fe-PA and MgO@Pp composites after adsorbing Cu^2+^ and Cd^2+^. The CS@Fe-PA and MgO@Pp composites were adsorbed, washed, and dried with deionized water multiple times to achieve the regeneration of CS@Fe-PA and MgO@Pp composites. Hydrochloric acid was chosen for eluting metal ions from adsorbents due to its widespread use in the industry, cost-effectiveness, and solubility of metal ions in it^94,95^. As depicted in Table 5, the removal efficiency dropped in subsequent cycles, reaching 97 to 92% for Cu^2+^ and 95.7% to 89.2% for Cd^2+^. In contrast, on CS@Fe-PA, the removal efficiency for Cu^2+^ and Cd^2+^ decreased from 90 to 81% and 87.2 to 68%, respectively, across the four regeneration cycles. Fig. 14a,b depicts that desorption efficiency decreased with each sorption-desorption cycle. The desorption efficiency of Cu^2+^ and Cd^2+^ was initially high for the first cycle, i.e., 94.6% and 90.9%, respectively. However, the performance of CS@Fe-PA declined, achieving desorption efficiencies of 87.3% for Cu^2+^ and 70.1% for Cd^2+^, which gradually decreased with subsequent cycles. The reduced efficiency of regenerated adsorbents may be attributed to the strong interaction between Cu^2+^ and Cd^2+^ and CS@Fe-PA and MgO@Pp composites. Water washing can only remove the adsorbates with low affinity to the surface of adsorbents, which are usually adsorbed by physical adsorption or weak chemical adsorption. Consequently, the number of active sites available on the surface of CS@Fe-PA and MgO@Pp composites decreased as the number of regenerations increased^8^.

**Table 5:** Comparing the reusability performance of CS@Fe-PA and MgO@Pp in removing metal ions with other nano-adsorbents.

Adsorbents	Metals	Cycles	Efficiency	Ref
Graphene oxide functionalized chitosan-magnetite	Cu (II)	Four cycle	86%	^5^
chitosan-modified polyethylene terephthalate	Cu	Four cycle	80 to 15%	^6^
ZnO@Chitosan	Cu(II), and Cd(II)	four cycle	90–96% and 90–95%	^7^
MgO@TiO2@g-C3N4	Cd(II)	Four cycle	83–98%	^8^
nano-calcium carbonate	Cu(II), and Cd(II)	Five cycle	80%	^9^
Fe3O4/graphene oxide	Cu(II), and Cd(II)	three cycle	97.39–90.52%	^10^
Orange peel	Cd(II)	One cycle	88.34–73.42%	^11^
magnetic activated car- bon	Cd(II)	Five cycle	90.5–80.5%	^12^
MgO@Pp	Cu(II), and Cd(II)	Four cycle	97–92% and 95.7–89.2%	This study
CS@Fe-PA	Cu(II), and Cd(II)	Four cycle	90–81% and 87.2–68%	This study

## Conclusions

The combination of different parent materials in the synthesis of composite adsorbents effectively merged their distinct properties, resulting in varying capabilities to remove Cu2+ and Cd2+ ions. XRD, SEM–EDX, and XPS characterization proved the formation of CS@Fe-PA and MgO@Pp composite materials. The effects of various experimental conditions (pH, adsorbent dosage, initial metal ion concentration and contact time) were examined. The SEM analysis confirmed the appropriateness of the material for the adsorption process, showcasing a rough surface and porous structure. The adsorption capacities of MgO@Pp showed much better sorption ability for Cu^2+^ and Cd^2+^ than the CS@Fe-PA. TGA demonstrated remarkable thermal stability of the synthesized MgO@Pp composite. The XPS analysis confirmed that the amino and hydroxyl groups chelated with metal ions*.* Thermodynamic parameters, including the change in free energy (ΔG°), enthalpy (ΔH°), and entropy (ΔS°), were also evaluated. The findings indicated an endothermic removal of Cu^2+^ and Cd^2+^ ions by MgO@Pp, whereas CS@Fe-PA demonstrated an exothermic process. Desorption studies on metal-adsorbed MgO@Pp and CS@Fe-PA composites were done with 0.2 M HCl and distilled water. This study revealed that mineral acid HCl was sufficiently helpful for the regeneration of metal ions from adsorbents. These results demonstrate that low-cost CS@Fe-PA and MgO@Pp composite adsorbents can be fabricated in a facile and environmentally friendly way, which are considerable candidates for adsorption of Cu^2+^ and Cd^2+^ ions from aqueous solutions.

## Supplementary Information

Supplementary Figure 1.

Supplementary Figure 2.

Supplementary Figure 3.

Supplementary Figure 4.

Supplementary Figure 5.

Supplementary Figure 6.

Supplementary Figure 7.

## Data Availability

All data generated or analyzed during this study are of our own work and it is our pleasure to be available on reasonable request. Connect with aurthor-mohamed_taha@nwrc.gov.eg.
