# Supplementary material for: Effective fabrication and characterization of eco-friendly nano particles composite for adsorption Cd (II) and Cu (II) ions from aqueous solutions using modelling studies
Source: Sci Rep. 2024 May 23;14:11767. doi: 10.1038/s41598-024-61050-1 (PMC11632089; doi:10.1038/s41598-024-61050-1)
Supplement: Supplementary file 3 — Supplementary Figure 3. [file 41598_2024_61050_MOESM3_ESM.docx]

a

**Y=2.254+33.12**

**R^2^=0.964**

b

**Y=2.36+30.57**

**R^2^=0.97**

c

d

**Y=0.587+33.1**

**R^2^=0.988**

Fig. 3 Kinetic adsorption results of Elovich model plots for the adsorption of Cu^2+^ and Cd^2+^ ions (a) ,(b) onto the CS@Fe-PA; and Cu^2+^ and Cd^2+^ ions (c),(d) onto MgO@Pp.
