# Supplementary material for: Effective fabrication and characterization of eco-friendly nano particles composite for adsorption Cd (II) and Cu (II) ions from aqueous solutions using modelling studies
Source: Sci Rep. 2024 May 23;14:11767. doi: 10.1038/s41598-024-61050-1 (PMC11632089; doi:10.1038/s41598-024-61050-1)
Supplement: Supplementary file 5 — Supplementary Figure 5. [file 41598_2024_61050_MOESM5_ESM.docx]

a

b

c

d

Fig. 5. Langmuir isotherm plots for the adsorption of Cu^2+^ and Cd^2+^ ions (a) ,(b) onto; CS@Fe-PA and Cu^2+^ and Cd^2+^ ions (c),(d) onto the MgO@Pp

d
