# Supplementary material for: Effective fabrication and characterization of eco-friendly nano particles composite for adsorption Cd (II) and Cu (II) ions from aqueous solutions using modelling studies
Source: Sci Rep. 2024 May 23;14:11767. doi: 10.1038/s41598-024-61050-1 (PMC11632089; doi:10.1038/s41598-024-61050-1)
Supplement: Supplementary file 6 — Supplementary Figure 6. [file 41598_2024_61050_MOESM6_ESM.docx]

a

b

c

d

Fig. 6 Temkin isotherm plots for the adsorption of Cu^2+^ and Cd^2+^ ions (a) ,(b) onto the CS@Fe-PA; and Cu^2+^ and Cd^2+^ ions (c),(d) onto MgO@Pp.
