# Supplementary figures and images for: Effective fabrication and characterization of eco-friendly nano particles composite for adsorption Cd (II) and Cu (II) ions from aqueous solutions using modelling studies
Source: Sci Rep. 2024 May 23;14:11767. doi: 10.1038/s41598-024-61050-1 (PMC11632089; doi:10.1038/s41598-024-61050-1)

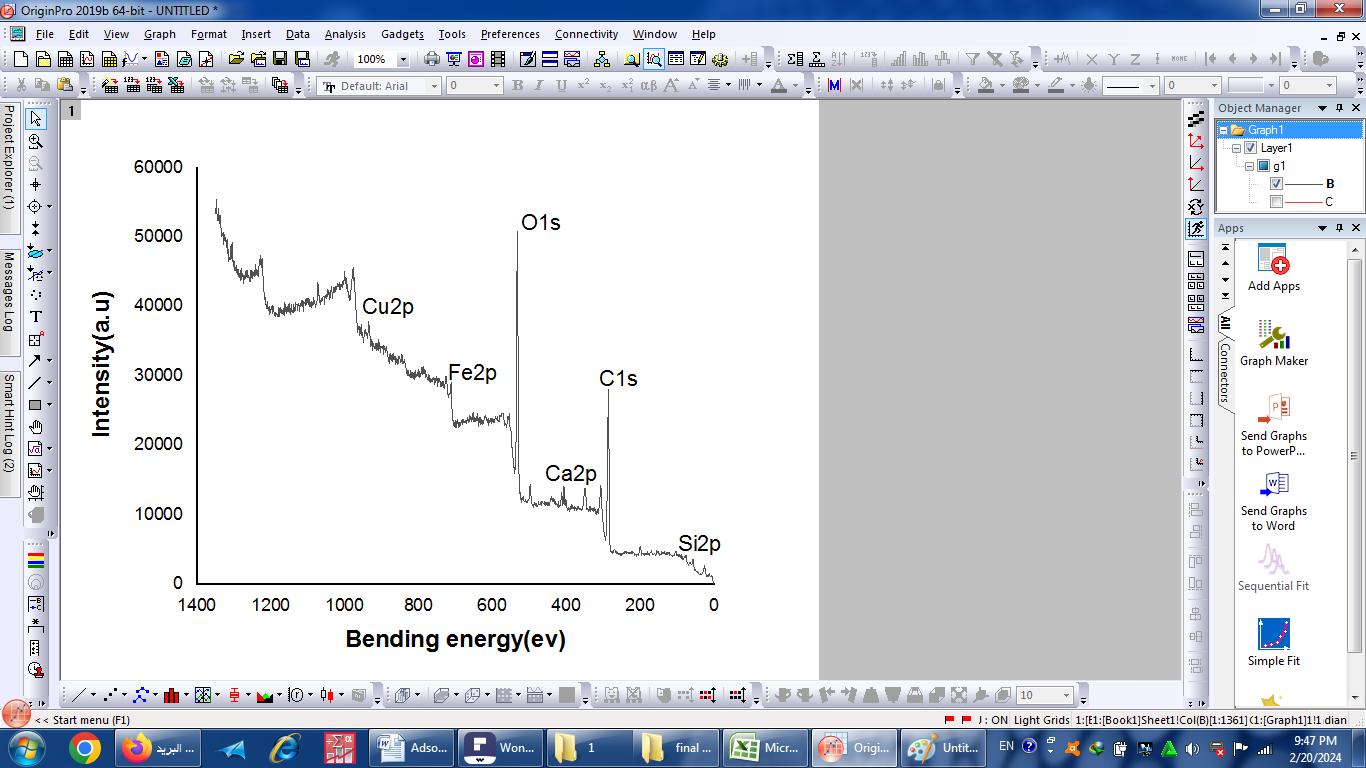

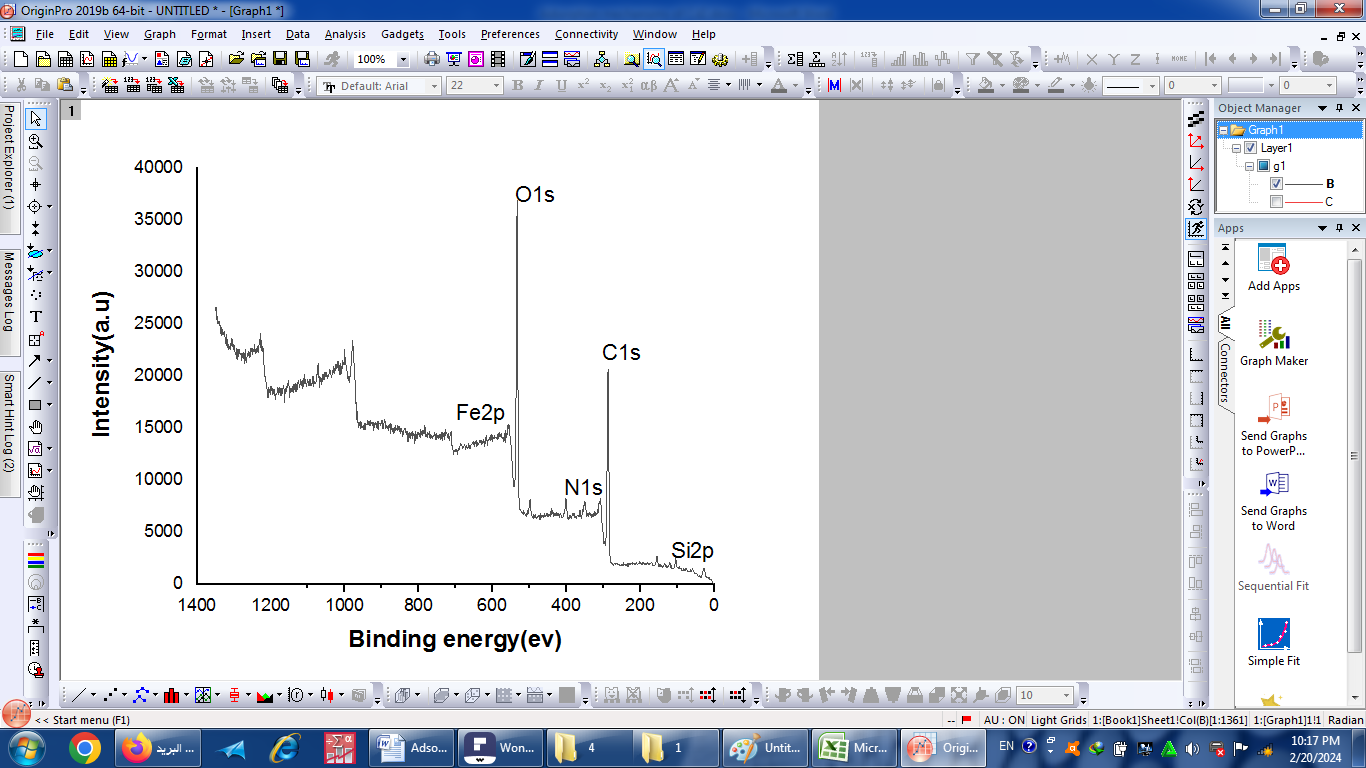


b

a

b


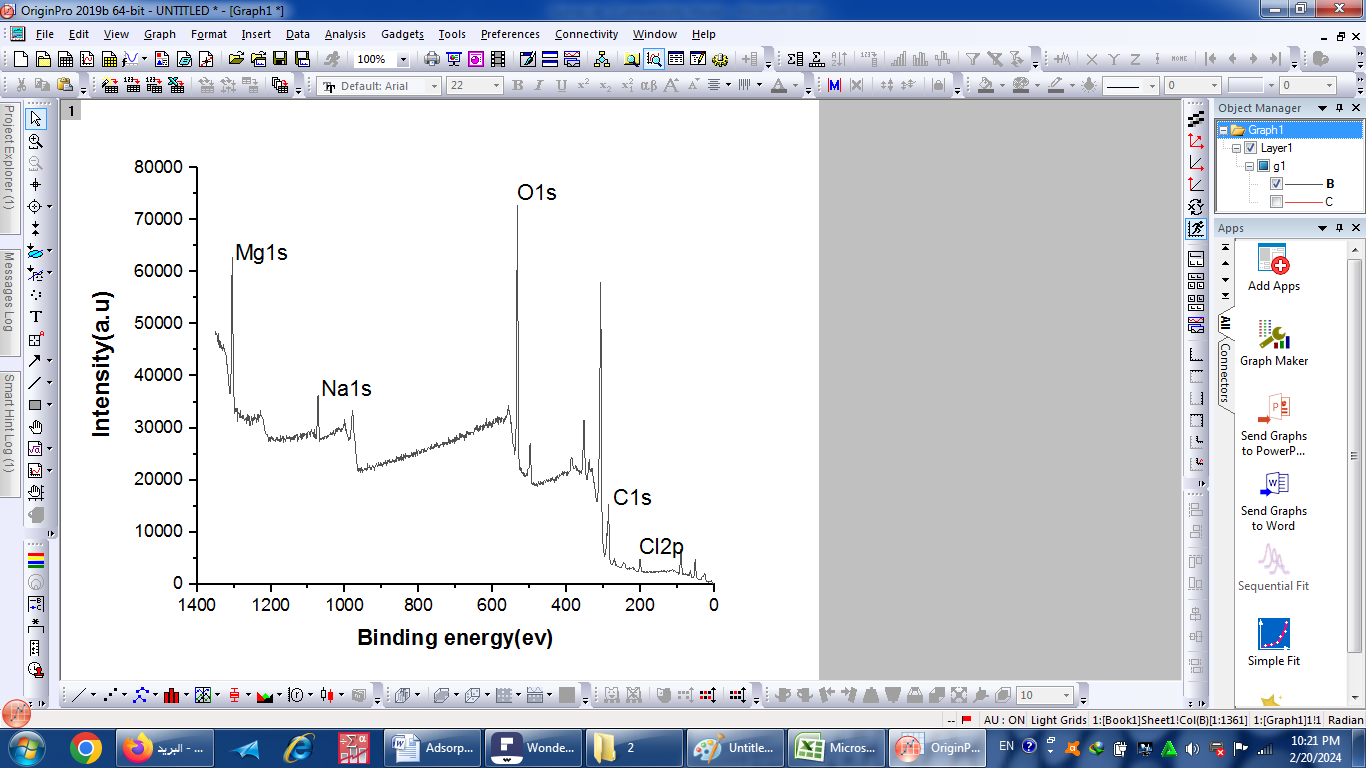


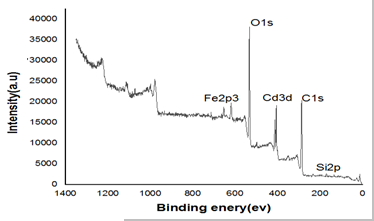


d

c


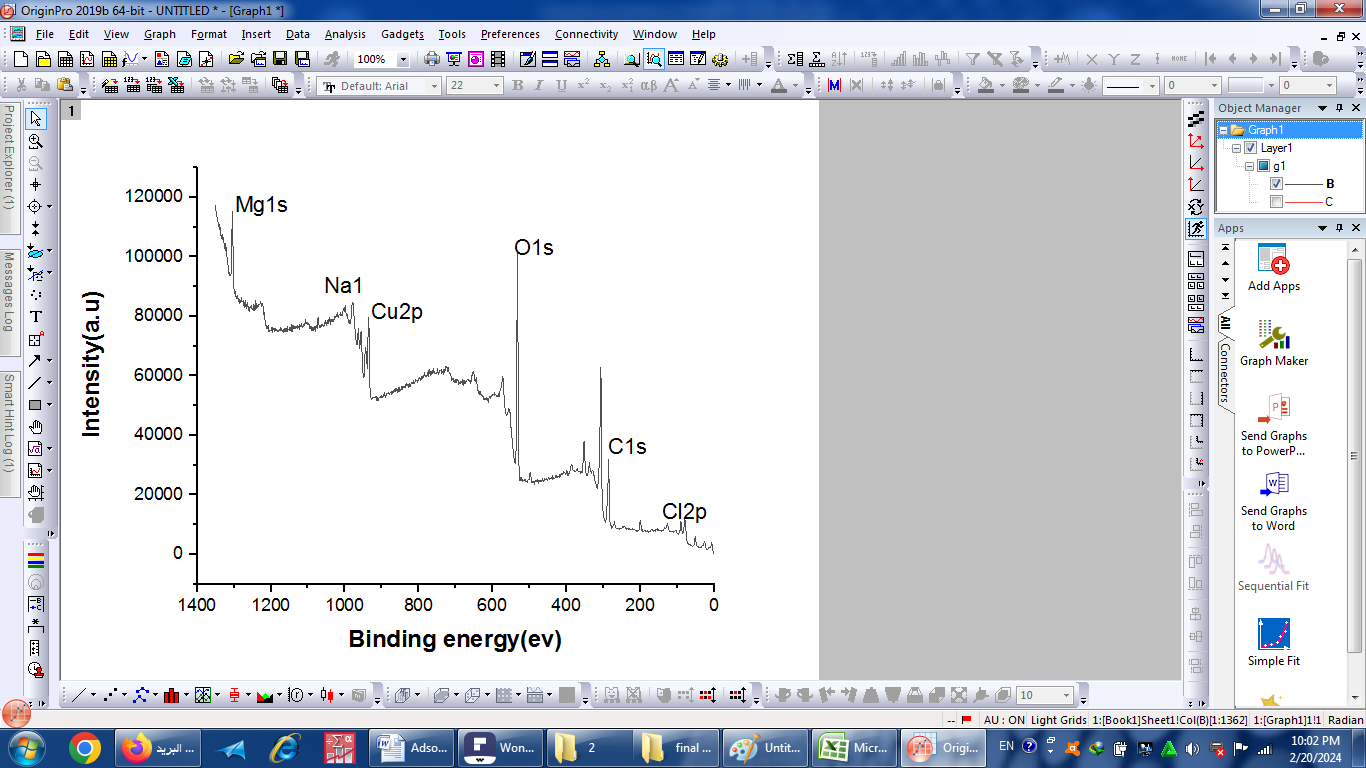


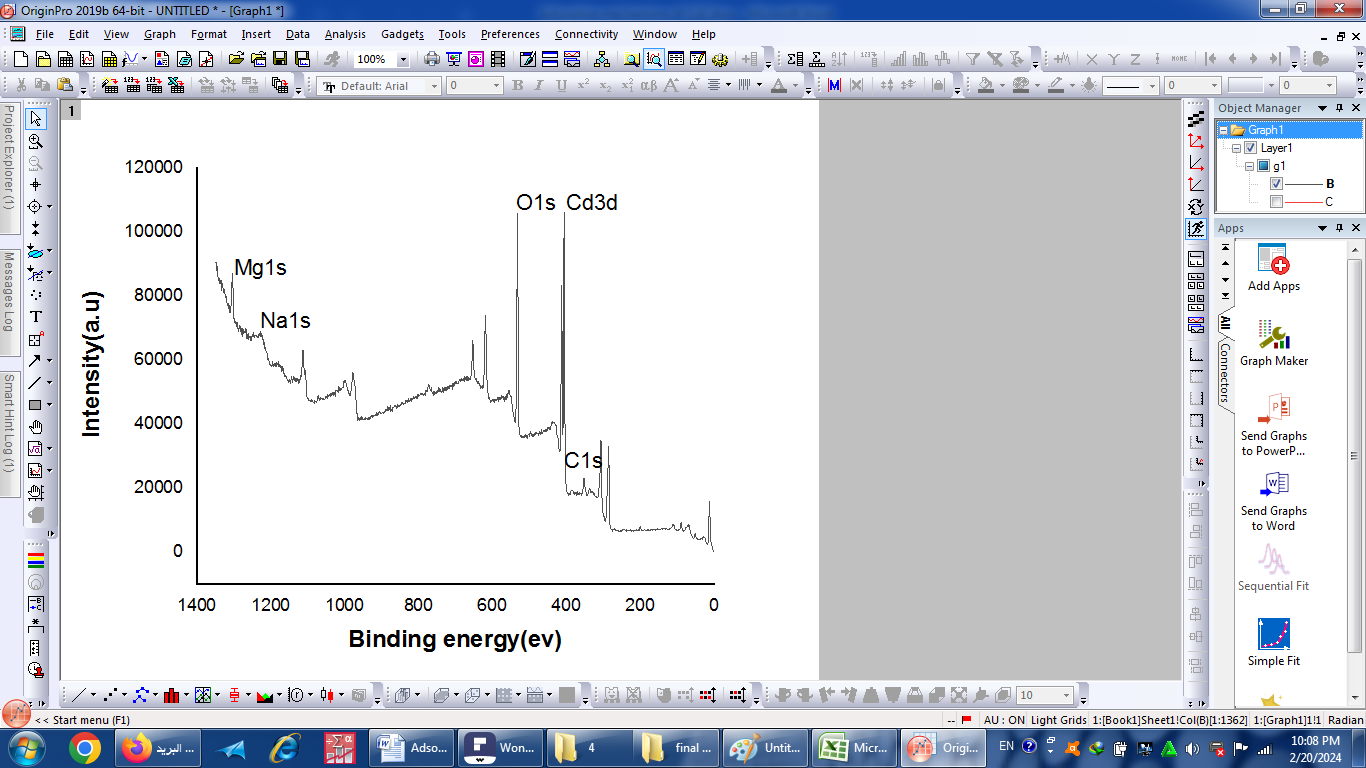


f

e

Fig. 7. (a) XPS images of (a)(b)(c)CS@Fe-PA and (d)(e)(f) MgO@Pp before and after Cd2+ and Cu2+ ions adsorption

Supplement: Supplementary file 7 — Supplementary Figure 7. [file 41598_2024_61050_MOESM7_ESM.docx]
